# HB52‐PUT2 Module‐Mediated Polyamine Shoot‐to‐Root Movement Regulates Salt Stress Tolerance in Tomato

**DOI:** 10.1111/pce.15479

**Published:** 2025-03-30

**Authors:** Xian Yang, Hongyi Qin, Yu Zhou, Ziqi Mai, Xirong Chai, Juxian Guo, Yunyan Kang, Min Zhong

**Affiliations:** ^1^ College of Horticulture South China Agricultural University Guangzhou China; ^2^ Vegetable Research Institute Guangdong Key Laboratory for New Technology Research of Vegetables, Guangdong Academy of Agricultural Sciences Guangzhou China

**Keywords:** HB52‐PUT2 regulatory module, polyamine long‐distance transport, salt tolerance, tomato

## Abstract

Soil salinity severely restricts crop quality and yields. Plants have developed various strategies to alleviate salinity stress's negative effects, including polyamine redistribution by polyamine uptake transporters (PUTs). However, the mechanisms by which PUTs alter polyamine translocation processes during salt stress have not been fully elucidated. Here, we show that disruption of *PUT2*, which is involved in polyamine shoot‐to‐root transport, results in salt sensitivity phenotypes in tomato. Moreover, yeast one‐hybrid screened for an HD‐Zip transcription factor *HB52* that interacts with PUT2, and loss of function of *HB52* also led to increased sensitivity to salt stress, whereas *HB52‐*overexpression lines exhibited improved salt tolerance. Furthermore, molecular analyses demonstrated that HB52 directly activated the expression of *PUT2* and facilitated Na^+^ efflux by promoting polyamine shoot‐to‐root mobility. This study uncovers a synergistic transcriptional regulatory network associated with a homeobox protein regulator that promotes polyamine long‐distance transport under salt stress.

## Summary statement

• This study reports that polyamine transport plays an important role in regulating salt tolerance in tomato.
• PUT2, a critical member of polyamine uptake transporters, is directly activated by the transcription factor HB52.
• Our results reveal that the HB52‐PUT2 module regulates polyamine mobility, enhancing salt tolerance.

## 1 Introduction

Soil salinization represents a challenging environmental condition that causes osmotic and oxidative damage and perturbs ion homoeostasis, limiting crop growth and decreasing crop productivity worldwide (Ismail and Horie 2017; Van & Testerink 2020). During evolution, plants have acquired various sophisticated mechanisms to adapt to salinity stress, including generating beneficial metabolites for cells and the organism as a whole. Polyamines are endogenous organic molecules that regulate, at a low level, various growth and development processes, and play a role in the stress response (Chen et al. 2019; Gerlin, Baroukh and Genin 2021). In plants, biogenic polyamines modulate development and dormancy, salt tolerance, lateral root emergence, and the production of reactive oxygen species under appropriate conditions, which are also activated in response to changing environment (Gémes et al. 2016; Yin et al. 2016).

Generally, multiple steps are involved in polyamine responses and functions: biosynthesis, transport, catabolism, perception, conjugation, and signal transduction (Fujita and Shinozaki 2014; Pál, Szalai and Janda 2021). Initially, putrescine is synthesised through the arginine decarboxylase (ADC) and ornithine decarboxylase (ODC)‐mediated pathways, and silenced ODC tomato lines exhibited an increase in *ADC* expression and polyamine levels, a compensatory effect of ADC while ODC reduction, but this compensatory effect was not observed in silenced *ADC* plants, a strong decrease in putrescine levels was observed in silenced *ADC* plants (Liu et al. 2015; González et al. 2022). In tomato, the ADC family consists of two members (ADC1 and ADC2), both of which have functions in stress response and development (Liu et al. 2024; Yang et al. 2024). Polyamines travel both short and long distances throughout in animals and plants, which is an essential feature discovered roughly ten years ago, enabling them to exhibit wide impacts in growth and responses to stimuli (Fujita and Shinozaki 2014; Vrijsen et al. 2020). It has been accepted that an optimal polyamine pool is required for their transportation to appropriate tissues and cells to elicit abiotic tolerance. However, recent studies have provided new evidence that polyamine transport is triggered by polyamine uptake transporters (PUTs). Nevertheless, the complete map of polyamine transport is incomplete, and the actual mechanism remains to be elucidated. Despite mounting evidence that several PUTs play a role in cellular transport in *Arabidopsis* (Li et al. 2013), rice (Lyu et al. 2022), and tomato (Zhong et al. 2023), the molecular mechanism underlying the long‐distance transport of polyamines during abiotic stresses, particularly salt stress, has not yet been reported.

In recent years, our understanding of polyamine pathway regulation has broadened to include polyamine transport and translocation. In the medical field, PUTs have been extensively studied for their involvement in diseases such as Parkinson's disease and cancer. The polyamine transporter improves mammalian lysosomal health and functionality by regulating polyamines from endocytic vesicles to the cytosol (Van Veen et al. 2020; Vrijsen et al. 2020). In *Escherichia coli*, PotABCD and PotFGHI were characterised as PUTs, and their homologues were identified in plants (Alhag et al. 2021; Stolarska et al. 2023; Thongbhubate et al. 2021). In botany, a spermidine‐specific transporter in rice (OsPUT1) was first identified (Mulangi et al. 2012). After that, the OsPUT2, OsPUT3.1, OsPUT3.2, and AtPUT1‐3 were determined to have polyamine uptake capacity in rice and *Arabidopsis*, respectively (Li et al. 2013; Mulangi et al. 2012). In our previous study, we confirmed the presence of PUT family members in tomato, and found that PUT2 was strongly associated with salt tolerance (Zhong et al. 2023). However, the upstream regulator that controls *PUT2* expression and the precise function of PUT2 in polyamine transport in response to salt stress remains elusive.

Determining the functions of polyamine largely depends on identifying the accurate roles of genes and their products in biotic and abiotic stress tolerance. Transcription factors (TFs), located upstream of functional genes, orchestrate the function of polyamine genes (Jiang et al. 2017; Pál, Szalai and Janda 2015). These complex components include a key interlinking module responsible for fine‐tuning various stress responses (Lan et al. 2017; Zhang 2003). Thus, deciphering the modules associated with known metabolic pathways is of tremendous value. Significant and meaningful work has been done to understand the regulatory modules involved in metabolites related to thousands of physiological or biological processes (Guo et al. 2022; Caffaro et al. 1993). For instance, many TFs acting as positive or negative regulators for metabolic synthesis have been discovered in plants, which has greatly advanced our in‐depth understanding of the regulatory networks (Joshi et al. 2016). To date, some TFs, including MYC2, CBF1, ABF3/4/30, NAC72 and WRKY70, have been suggested to likely govern the expression of polyamine‐related genes and contribute to polyamine accumulation in response to multiple stresses (Gong et al. 2015; Liu et al. 2022; Liu et al. 2022; Min et al. 2021; Song et al. 2023). However, the mechanism of how TFs meditate polyamine translocation and associated responses under salt conditions remains unclear.

The plant‐specific leucine zipper‐homeodomain Ⅰ (HD‐Zip Ⅰ) TF family is a subfamily of homeobox proteins, consisting of seventeen members in *Arabidopsis*, many of which are known to participate in different developmental and growth processes (Ariel et al. 2007). Various hormonal and environmental signals can induce many HD‐Zip Ⅰ genes. For instance, drought and salinity stress can activate the Arabidopsis AtHB7 and AtHB12 genes (Ré et al. 2014). In Chickpea, *CaHDZ12* overexpression lines display a soluble sugar‐ and proline‐overproduced phenotype, indicating a function of CaHDZ12 in sugar and proline‐mediated salt stress tolerance (Sen et al. 2017). Moreover, the Populus PttHB8 was suggested to play an important role in the accumulation of polyamines by regulating the expression of the thermospermine synthase gene *ACAULIS5* (Milhinhos et al. 2013). However, the functional role of HD‐Zip TFs in the control of polyamine transport and in inducing salt tolerance is largely unknown.

To explore these factors and elucidate the underlying mechanism of the role of PUTs in salt stress tolerance in plants, we performed loss‐of‐function, overexpression, and grafting experiments in tomato, focusing on PUT2. Loss‐of‐function studies showed that *PUT2* is required for salt tolerance by limiting Na^+^ influx. In addition, a grafting and polyamine transport experiment confirmed that *PUT2* is crucial for long‐distance polyamine translocation from the shoot to the root. To further explore the upstream regulatory mechanism by which PUT2 acts as a key regulator in response to salt stress, we discovered that the HD‐Zip TF homeobox 52 (HB52) binds directly to the promoter of *PUT2*. Furthermore, we established tomato lines with loss of function and overexpression of the *HB52*, which has recently been reported to regulate phytohormone and the responses to abiotic stress (Ariga et al. 2022; Miao et al. 2018). *HB52*‐overexpression plants showed salt stress tolerance, while loss‐of‐function *hb52* mutants showed salt‐sensitive phenotypes. Therefore, this study provides a new mechanism by which the HB52‐PUT2 module activates polyamine long‐distance transport to respond to salinity conditions.

## 2 Results

### 2.1 Polyamine Shoot‐to‐Root Transport Mitigates Salt Stress

**Figure 1:**
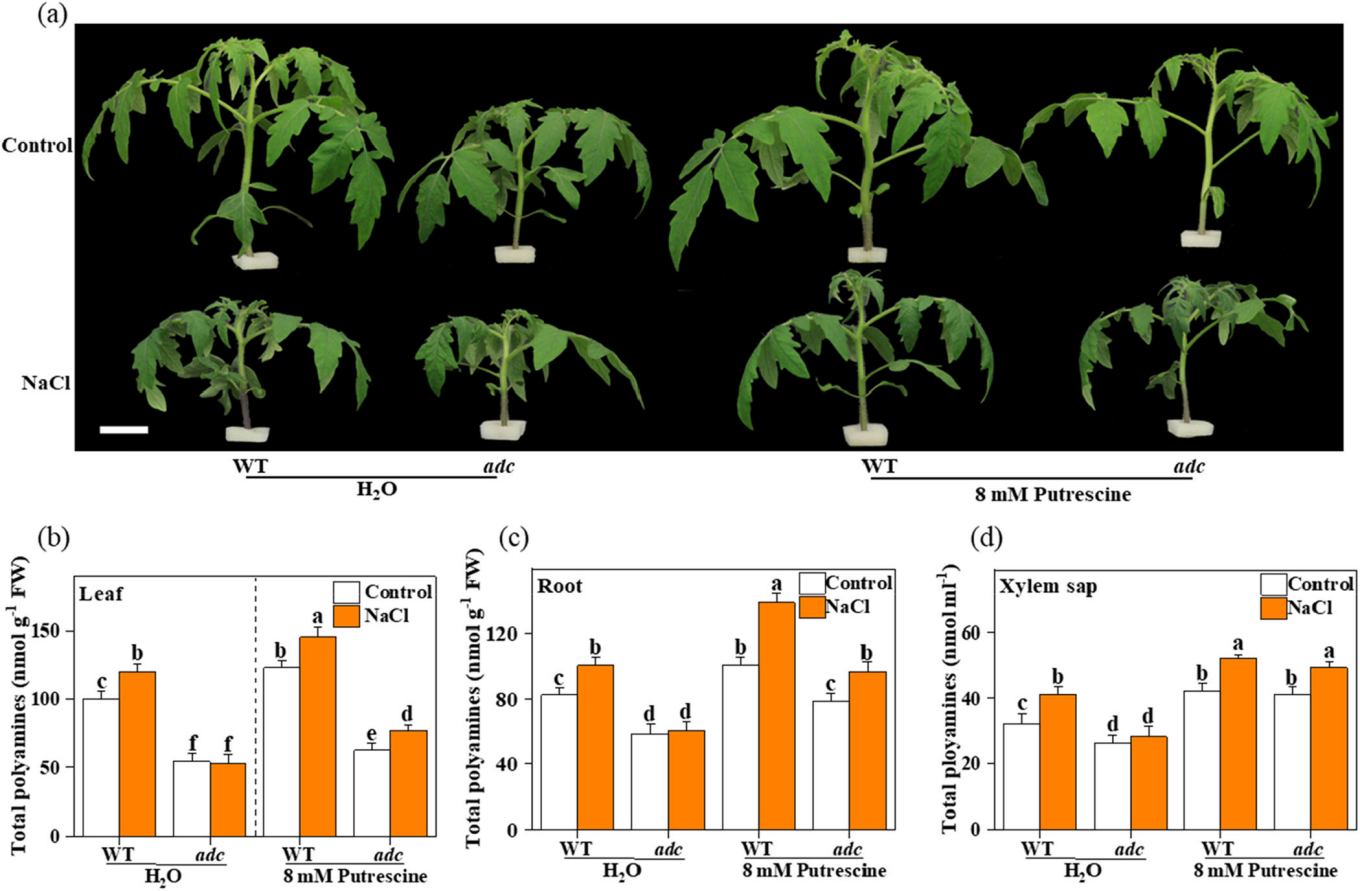
Effect of long‐distance polyamine transport on the phenotype of WT and putrescine biosynthetic mutants (*adc1*) of tomato under salt stress. (a) Phenotypes of exogenous putrescine‐mediated salt tolerance in WT and *adc1* mutant, photographs were taken 7 days after treatment, Bar = 5 cm; (b–d) Total polyamines content in leaf (b), root (c), and xylem sap (d) in WT and *adc1* mutant after exogenous putrescine treatment under salt stress for 7 days. Data are presented as mean values ± SD; *n* = 3. Different letters indicate significant treatment differences (*p* < 0.05, Duncan's multiple range test). At least three independent experiments were performed.

Polyamine impacts plant salt response by modulating the related defence pathways. Still, it has not been characterised in the systematic shoot‐to‐root mobility of polyamine of abiotic stress responses in plants. Therefore, to determine the shoot‐to‐root mobility of polyamine under salt conditions, we focused on the role of ADC. Due to the inability of the tomato *adc1/2* mutant to produce seeds, and the significant reduction of putrescine content in tomato *ADC1*‐VIGS plants (Wu et al. 2019; Kundu et al. 2022), we generated an *adc1* CAS9 knockout allele that contained a deletion in the coding sequence using the clustered regularly interspaced short palindromic repeat (CRISPR)‐associated protein9 (Cas9) genome editing system (Figure S1). Tomato plants homozygous for the *adc1* allele showed higher sensitivity to salinity stress compared with the wild‐type (WT), as evidenced by increased relative electrolyte leakage (REL) and reduced the maximum quantum yield of photosystem II photochemistry (*F*_*v*_/*F*_*m*_) after 7 days in salt (120 mM NaCl) conditions (Figure S2). We also found that the endogenous polyamine accumulation under normal or salinity conditions evident in WT plants was weakened in this mutant, which was associated with the relatively shorter phenotype of the *adc1* mutant (Figure 1). We further tested the response of the *adc1* mutant to salt stress in the presence of putrescine. Exogenous application of 8 mM putrescine significantly affected the response of the *adc1* mutant to salinity stress; it markedly rescued the salt‐sensitive phenotype of the *adc1* mutant and restored the dwarfing phenotype under normal conditions (Figure 1a and Figure S2). Furthermore, following the application of putrescine to the surface of the leaves, high‐performance liquid chromatography (HPLC) confirmed that polyamines accumulated in the leaf, root, and xylem sap of the *adc1* mutant (Figure 1b–d); these results indicated that polyamine shoot‐to‐root mobility is critical for conferring salt tolerance in tomato.

### 2.2 PUT2 Regulates Long‐Distance Polyamine Transport to Limit Na^+^ Influx

**Figure 2:**
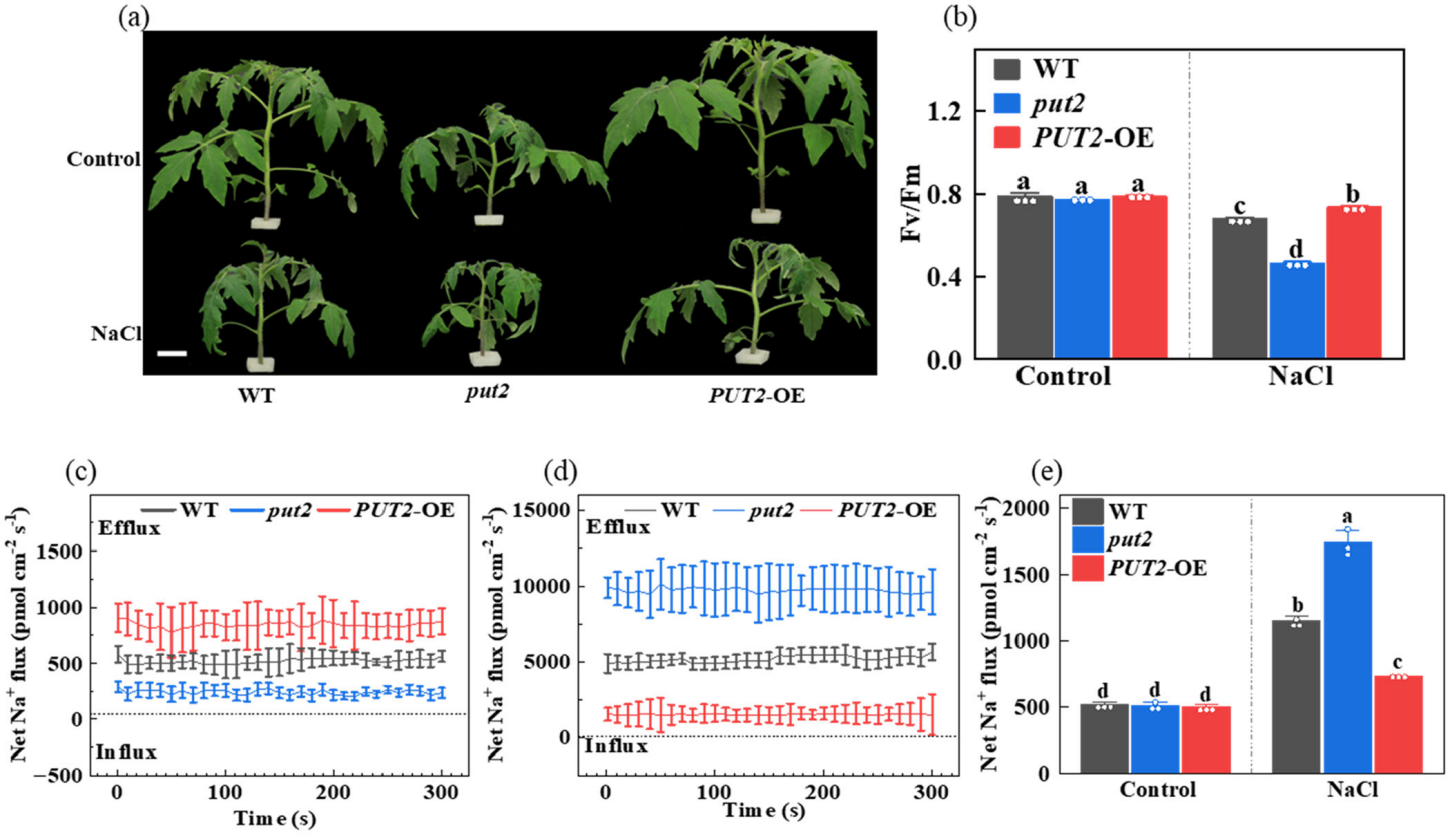
Polyamine uptake protein 2 (PUT2) positively regulates salt tolerance. (a) Phenotypes of WT, *put2* mutant and *PUT2*‐overexpression (*PUT2*‐OE) plants exposed salt treatment for 7 days, Bar = 5 cm; (b) The Fv/Fm in WT, *put2* mutant, and *PUT2*‐OE plants exposed to salt treatment for seven days; (c) Net Na^+^ fluxes in 500 μm distance from root apex of WT, *put2* mutant, and *PUT2*‐OE plants under salt treatment; (d) Net Na^+^ fluxes of the root xylem parenchyma cell in the roots of WT, *put2* mutant, and *PUT2*‐OE plants under the treatment of 120 mM NaCl for 24 h; (e) Quantitative analysis of the means of net Na^+^ fluxes within a continuous period of 0–5 min. Data are presented as mean values ± SD; *n* = 3. Different letters indicate significant treatment differences (*p* < 0.05, Duncan's multiple range test). At least three independent experiments were performed. [Color figure can be viewed at wileyonlinelibrary.com]

Given our recent finding that PUT2 plays a positive role in salt stress, and polyamine transport in yeast, to further explore its function in the salt response and the underlying mechanism, PUT2‐knockout mutant (*put2*) and *PUT2*‐overexpressing (*PUT2*‐OE) tomato plants were established (Zhong et al. 2023). The *put2* mutant displayed significant reductions in shoot growth compared to the WT (Figure 2a). After exposure to high‐salt conditions for 7 days, the *PUT2*‐OE seedlings exhibited an increased salt tolerance phenotype, whereas the *put2* mutant was more sensitive to salt stress (Figure 2a). Consistently, the *PUT2*‐OE seedlings showed reduced REL and increased *F*_*v*_ */F*_*m*_, whereas the *put2* mutant displayed higher REL and decreased *F*_*v*_/*F*_*m*_ compared to those of the WT (Figure 2b and Figure S3). Furthermore, we measured the Na^+^ effluxes in WT, *put2* mutant, and *PUT2*‐OE plants using noninvasive microtest (NMT) technology. Salt stress induced an obvious fluctuation of Na^+^ flux in the roots of the WT, *put2* mutant, and *PUT2*‐OE plant, with the *PUT2*‐OE line exhibiting significantly higher Na^+^ efflux upon salt treatment throughout the measuring period than that of the WT. In addition, *put2* mutant roots showed lower Na^+^ efflux than those of the WT and *PUT2*‐OE (Figure 2c). Moreover, NMT measurements exhibited that compared with the WT, the Na^+^ flux of xylem parenchyma cells cut from the primary root was markedly higher in *put2* mutants and lower in *PUT2*‐OE plants (Figure 2d). In the presence of salt treatment, the *PUT2*‐OE roots had higher net Na^+^ efflux, which significantly decreased during salt stress, and was much lower in the *put2* mutant relative to that in the WT (Figure 2e). Moreover, a significant accumulation in the levels of spermidine, putrescine, and spermine was observed in *PUT2*‐OE plants, while the content in the *put2* mutant was lower compared with WT plants in the presence or absence of salt stress (Figure S4). Furthermore, the function of PUT2 in polyamine transport in plants was determined by analyzing polyamine uptake in the *put2* mutant compared with that in the WT. The *put2* mutant seedlings showed significantly lower ^13^C‐spermidine uptake rate and ^13^C‐Spd concentration in roots with respect to that in the WT in the foliar application of 2 μm ^13^C‐spermidine (Figure S5a). These results indicated that the PUT2 is a shoot‐to‐root transporter for polyamines.

**Figure 3:**
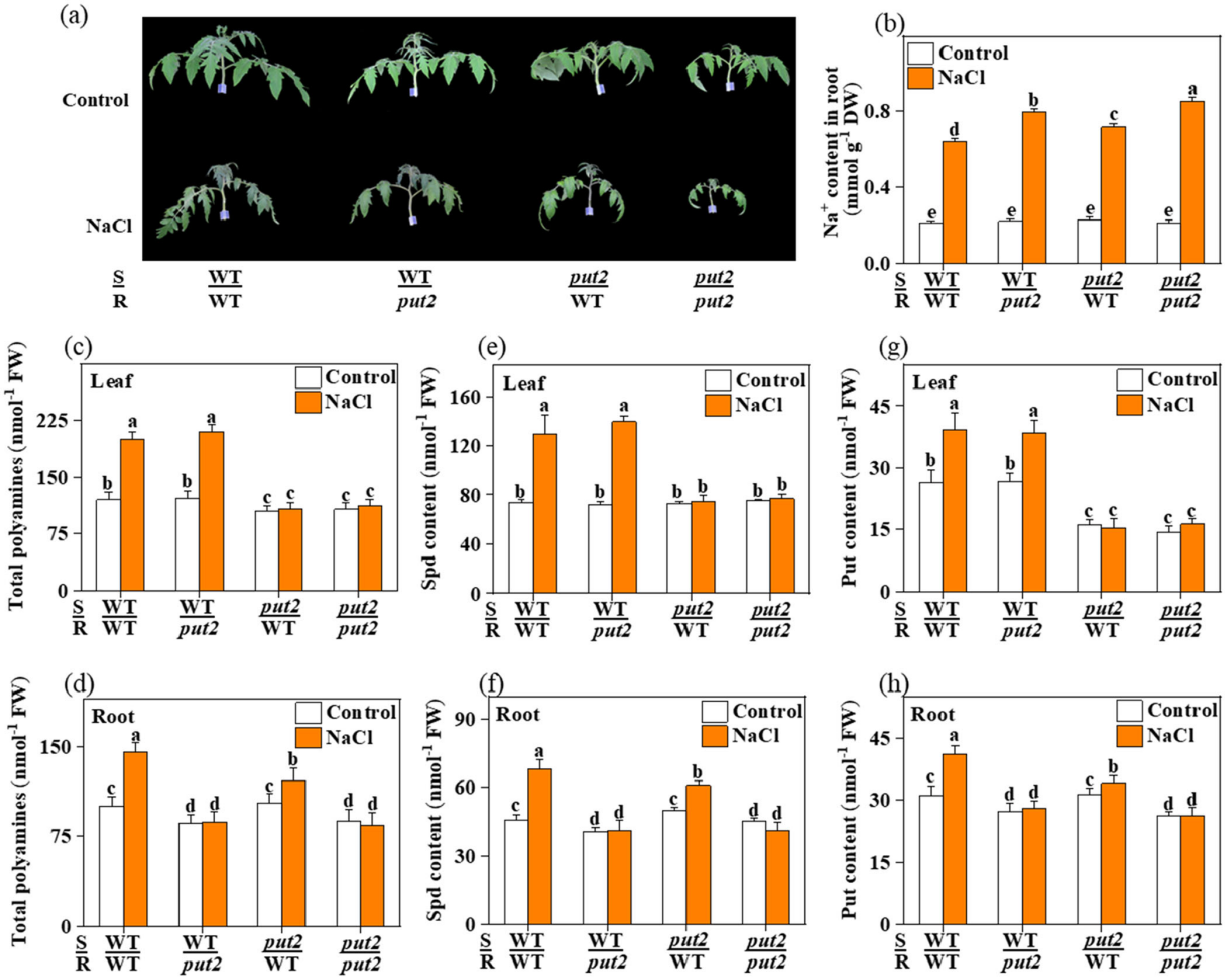
PUT2‐dependent shoot‐to‐root polyamine transport is essential for salt resistance. (a) Phenotypes in grafted plants with *put2* as rootstock or scion with or without salt treatment for 7 days; (b) The Na^+^ content in root in grafted plants with *put2* as rootstock or scion exposed salt treatment for 7 days; (c, e, and g) Total polyamines, spermidine (Spd), and putrescine (Put) accumulation in leaf in grafted plants with *put2* as rootstock or scion with or without salt treatment for 7 days; (d, f and h) Total polyamines, Spd, and Put accumulation in root in grafted plants with *put2* as rootstock or scion with or without salt treatment for 7 days; Data are presented as mean values ± SD; *n* = 3. Different letters indicate significant treatment differences (*p* < 0.05, Duncan's multiple range test). At least 3 independent experiments were performed. [Color figure can be viewed at wileyonlinelibrary.com]

To provide more evidence of the role of PUT2 in the systematic shoot‐to‐root mobility of polyamine, reciprocal graft combinations of the WT and *put2* mutant plants were generated: WT/WT, WT/*put2*, *put2*/WT, and *put2*/*put2* (scion/rootstock), which were then subjected to salt treatment. Plants with *put2* as the rootstock (WT/*put2* and *put2/put2*) or scion (*put2*/WT) displayed decreased resistance to salt treatment compared to that of the WT/WT (S/R) plants, as evidenced by decreased *F*_*v*_/*F*_*m*_ and increased Na^+^ content in the root (Figure 3a,b and Figure S5b). The WT/WT and WT/*put2* plants exhibited better above‐ground growth, and had higher polyamine accumulation in the leaves compared to those of the *put2*/WT and *put2*/*put2* plants, and the WT/WT and *put2*/WT plants exhibited increased levels of polyamines in the roots relative to those of WT/*put2* and *put2*/*put2* plants under non‐stress conditions (Figure 3a,c,d), it may be that grafting impaired polyamine shoot‐to‐root transport. Crucially, although salt treatment induced remarkable increases in the accumulation of polyamines in the leaves of WT/WT and WT/*put2* plants, as well as in the roots of WT/WT and *put2*/WT plants, these effects were either attenuated or not evident in the *put2*/WT leaves and the WT/*put2* and *put2*/*put2* roots (Figure 3c,d). In addition, the spermidine and putrescine contents showed similar trends in these graft chimeras, but there was no significant difference in the spermine content between with and without salt stress conditions (Figure 3e–h and Figure S6). Together, these observations indicated that PUT2 is induced by salt stress to transport polyamines from the shoot to the root to attenuate salt‐induced damage.

### 2.3 HB52 Positively Regulates Salt Tolerance

**Figure 4:**
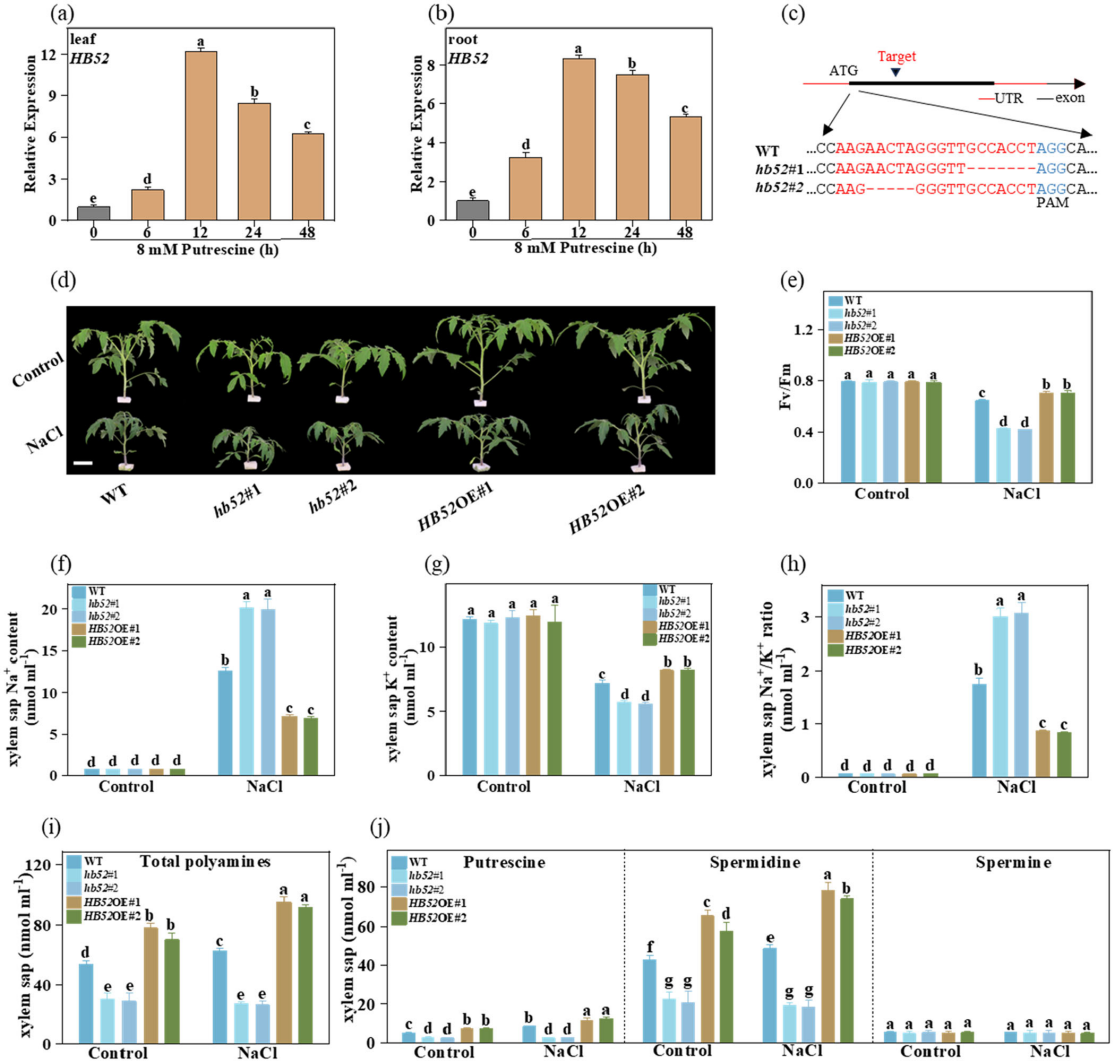
HB52 is upregulated under salt stress conditions. (a, b) Time course analysis of putrescine‐induced *HB52* expression of leaf (a) and root (b) in WT seedlings; (c) Generation of *hb52* mutants by CRISPR‐Cas9 system. Blue letters indicate protospacer‐adjacent motif (PAM) sequences, and the red dash indicates deletion; the *hb52*#1 line has a seven bp deletion, and the *hb52*#2 line has a five bp deletion, leading to early termination of HB52 protein, respectively; (d) Phenotypes of WT, *hb52* mutant and *HB52*‐overexpression (*HB52*‐OE) plants exposed salt treatment for 7 days; Bar = 5 cm; (e) The Fv/Fm in WT, *hb52* mutant, and *HB52*‐OE plants exposed to salt treatment for seven days; (f, g and h) The Na^+^, K^+^ and Na^+^/K^+^ ratio in xylem sap in WT, *hb52* mutant and *HB52*‐OE plants exposed salt treatment for 7 days; (i) The total polyamines content in xylem sap in WT, *hb52* mutant, and *HB52*‐OE plants exposed to salt treatment for seven days; (j) The individual polyamines putrescine, spermidine, and spermine levels of the same samples are shown in j. Data are presented as mean values ± SD; *n* = 3. Different letters indicate significant treatment differences (*p* < 0.05, Duncan's multiple range test). At least three independent experiments were performed. [Color figure can be viewed at wileyonlinelibrary.com]

We found that putrescine, one of the most important polyamines, has vital roles in enhancing salt tolerance and can induce the expression of *HB52* (Figure 4a,b). Hence, to investigate whether HB52 plays a role in conferring halotolerance in response to the accumulation of polyamines, we established two CAS9 mutant lines (*hb52*#1 and *hb52*#2) and two overexpressing lines (*HB52*‐OE#1 and *HB52*‐OE#2) in tomato (Figure 4c and Figure S7). The two gene‐edited lines (*hb52*#1 and *hb52*#2) produced by the CRISPR‐Cas9 system in the WT background had a 7‐bp or 5‐bp deletion of the exon of HB52, which resulted in a truncation of the HB52 protein (Figure 4c). Morphological observations showed that the *hb52* mutants have a dwarf phenotype than the WT plants. WT plants were significantly smaller than the *HB52*‐OE plants under control conditions (Figure 4d), which may reflect the perturbations in polyamines and hormones. Furthermore, Phenotype analysis showed that both lines of *HB52*‐OE plants displayed salt tolerance compared to the WT plants. In contrast, the *hb52* mutants showed impaired salt tolerance, with a lower Fv/Fm and higher REL than the WT (Figure 4d,e and Figure S8). These results suggested that HB52 is a positive regulator of salt tolerance.

Given its reported crucial role in regulating the efflux of excess Na^+^ from cells and facilitating the long‐distance transport of Na^+^ in plants in response to salt stress (Zhu 2002), we next investigated whether HB52 plays a role in Na^+^/K^+^ homoeostasis or transport. Without salt treatment, the WT, *hb52*, and *HB52*‐OE leaves and roots had similar Na^+^ and K^+^ contents (Figure S9). However, when the plants were subjected to salt stress, the Na^+^ content increased significantly in the roots and shoots in *hb52* mutants, whereas the salt‐induced Na^+^ accumulation was reduced in *HB52*‐OE plants. The *hb52* mutants displayed lower K^+^ content in their roots and shoots than *HB52*‐OE plants under salt stress (Figure S9).

To determine whether this HB52‐reduced Na^+^ accumulation in the shoots was due to its transport from the roots, we further analyzed the levels of Na^+^ and K^+^ in the xylem. Na^+^ accumulated in the xylem sap of WT plants under salt stress, with substantially greater accumulation in the *hb52* mutants, and reduced levels in *HB52*‐OE plants (Figure 4f). Salt treatment also led to a reduced K^+^ concentration in the xylem sap of the *hb52* mutant, whereas a much higher K^+^ content was found in the *HB52*‐OE xylem sap compared with that of the WT (Figure 4g). Consistently, the Na^+^/K^+^ ratio of the xylem sap was higher in *hb52* and lower in *HB52*‐OE than that of the WT (Figure 4h).

The effects of polyamine on salt tolerance partially depend on shoot‐to‐root polyamine transport (Figure 3). Therefore, we tentatively speculated that HB52 may function in polyamine transport. Compared to the WT, the total polyamines level decreased in the *hb52* mutants' xylem sap and increased in the *HB52*‐OE plants under normal conditions. Under salt stress, the total polyamine content in the *hb52* mutant was significantly lower than that in the WT, whereas it substantially increased in the *HB52*‐OE lines (Figure 4i). Furthermore, putrescine and spermidine were the polyamines with the highest abundance in the xylem sap after salt treatment, and salt stress did not result in variation in the spermine content in the xylem sap among the three genotypes (Figure 4j). These findings suggested that HB52 plays a critical role in the shoot‐to‐root polyamine transport and balances the Na^+^/K^+^ ratio in response to salt stress.

### 2.4 HB52 Is a TF That Positively Regulates *PUT2* Expression

**Figure 5:**
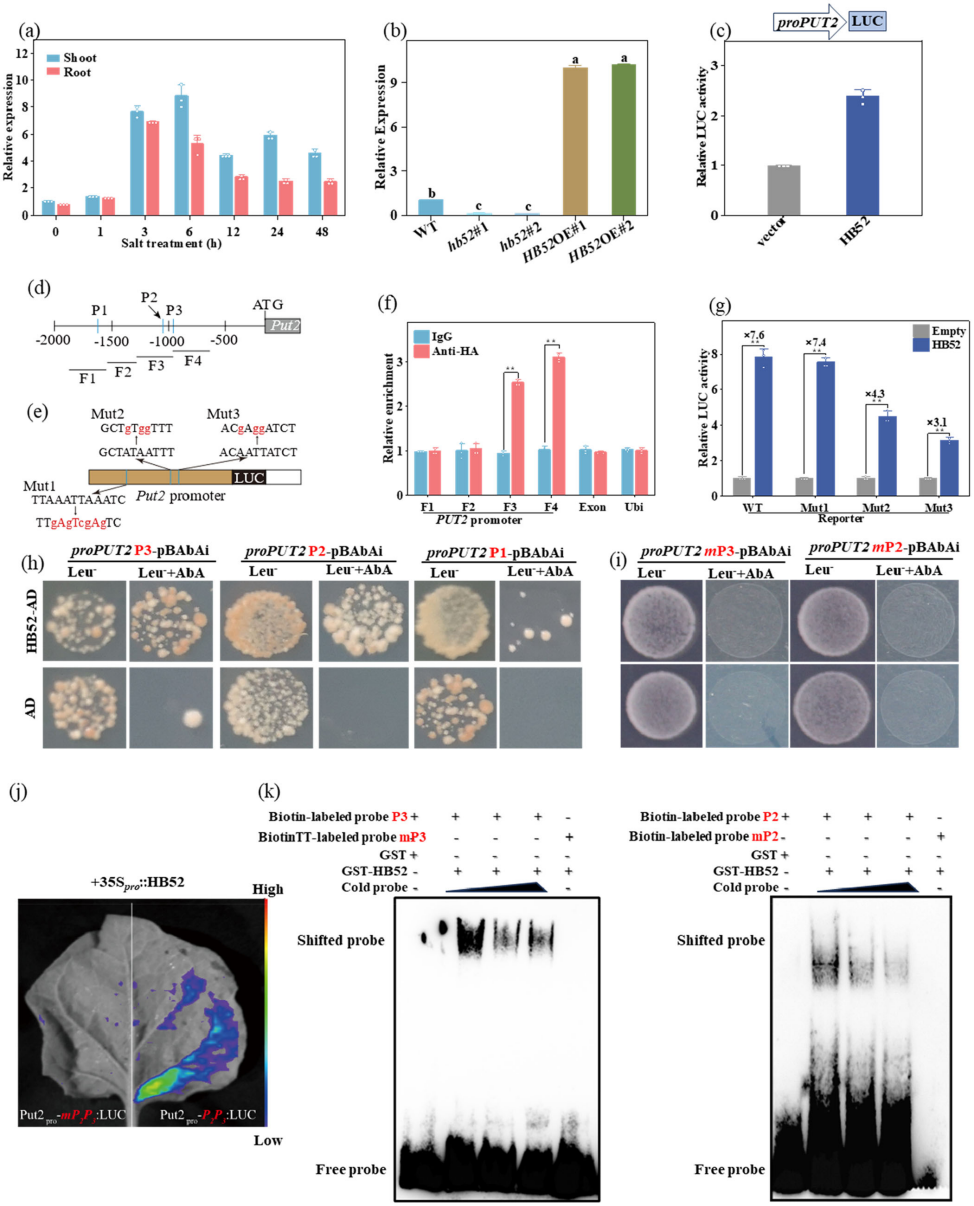
HB52 positively regulates salt tolerance by activating *PUT2* expression. (a) Relative expression of *PUT2* in leaves and roots. Three‐week‐old seedlings were treated with or without 120 NaCl for 48 h; (b) Expression levels of *PUT2* in three‐week‐old WT, *hb52* mutans, and *HB52*‐OE plants; (c) Transient expression of HB52 in tobacco leaves activates *PUT2* promoter activity; (d) Schematic diagram of *PUT2* promoter. Vertical blue bars indicate putative HB52‐binding sites. Grey horizontal lines represent the PCR fragments (F1–F4) for the ChIP assays in f. Black horizontal lines represent the probes (P1–P3) for the Y1H assays in (h); (e) Reporter constructs for cotransfection assays. Red lowercase letters represent nucleotide substitutions introduced into the consensus sequence to generate mutated *PUT2* promoter and mutant EMSA probe; (f) ChIP‐qPCR assays indicate that HB52 binds to the *PUT2* promoter in vivo. The data show relative enrichment of DNA precipitated with Flag antibody to those treated with IgG (set to 1). The fragments in an exon of *PUT2* and promoter of Ubi were used as controls. Data are means ± SD (*n* = 3); (g) Dual‐luciferase assay for the regulatory effect of HB52 on the expression of *PUT2*. WT and mutated *PUT2* promoter were used for the assay. The ratio of LUC/REN of the empty plus promoter was set as one; (h) Yeast one‐hybrid (Y1H) experiment showing the binding of HB52‐AD to the P2 or P3 regions of the *PUT2* promoter; (i) Y1H experiment shows HB52 could not bind to the mutated P2 or P3 regions of the *PUT2* promoter. (j) Dual‐luciferase assay shows HB52 could not bind to the mutated P2P3 regions of the *PUT2* promoter; (k) EMSA assay. The HB52:GST recombinant protein was incubated with biotin‐labelled WT (PUT2‐P2‐wt, PUT2‐P3‐wt) or mutant (PUT2‐P2‐m, PUT2‐P3‐m) oligos. The protein purified from the empty vector was used as a negative control. In (b), data represent the mean ± SD of three biological replicates. Asterisks (**p < 0.01; Student's *t*‐test) indicate significant differences identified between WT plants and other samples (f), between samples using HB52 expression and empty plasmids (g). [Color figure can be viewed at wileyonlinelibrary.com]

Reverse transcription‐quantitative polymerase chain reaction (RT‐qPCR) was performed to examine the *HB52* transcript patterns in different tissues; relatively high expression levels of HB52 were detected in leaves, seeds, and flowers (Figure S10). Similarly, in Arabidopsis, relatively high expression levels of *HB52* were detected in the above‐ground tissues, including in roots, stems, and rosette leaves (Miao et al. 2018). *HB52* expression was sustainably induced by salt stress (Figure 5a); and transiently induced by treatment with methyl jasmonate and abscisic acid (Figure S10). Furthermore, the *PUT2* transcript levels were reduced in *hb52*#1 and *hb52*#2 mutants but increased in the *HB52*‐OE lines, suggesting HB52 regulates *PUT2* expression (Figure 5b). In addition, a dual luciferase (LUC) assay showed that transfection of the HB52‐HA construct significantly increased *proPUT2*::LUC activity in tobacco leaves, indicating that HB52 induces *PUT2* promoter activity in vivo (Figure 5c).

As a member of the HD‐Zip I subfamily, HB52 can bind to the CAAT(A/T)ATTG sequence (Ariel et al. 2007). Promoter sequence analysis confirmed three putative HD‐binding sequences within the 2000‐bp promoter region of *PUT2*. Among these, we designed four fragments, designated F1–F4: F1, F3, and F4 contained at least one HB52 potential binding sequence (Figure 5d). Chromatin immunoprecipitation (ChIP)‐qPCR analysis showed that HB52 strongly bound two fragments (F3 and F4) at the PUT2 promoter. Subsequent cotransfection analysis in tobacco leaves revealed that HB52 activates WT *PUT2* promoter‐dependent transcription, but shows different effects on mutant *PUT2* promoter‐dependent transcription (Figure 5e–g). A comparison of the activation levels (fold change) among *PUT2* promoters suggested that only the F3 and F4 putative binding sites in the *PUT2* promoter are functional for interaction with HB52.

Similarly, a yeast one‐hybrid assay showed that yeast cells containing only the bait pAbAi vector harbouring the P2 and P3 *PUT2* promoter regions but not the P1 region grew on the selection medium (Leu^‐^+AbA) when co‐transformed with HB52‐AD, whereas those transformed with the empty pGADT7 vector did not grow on the Leu^‐^+AbA medium. The bait vector harbouring mutated P2 and P3 *PUT2* promoter regions did not grow on the Leu^‐^+AbA medium when co‐transformed with HB52‐AD in yeast cells (Figure 5e,h,i). The fluorescence intensity of the *PUT2*_pro_‐P2P3::LUC complex was substantially activated in the presence of 35S_pro_::HB52, whereas the *PUT2*_pro_‐mP2P3::LUC complex exhibited very low expression in tobacco leaves (Figure 5j).

Based on these results, we further focused on the P2 and P3 *PUT2* promoter regions as the candidate sites for interaction with HB52. In vitro electrophoretic mobility shift assays (EMSAs) showed that biotin‐labelled probes (P2 and P3) caused a mobility shift in the F3 and F4 fragments, whereas the mutation probes did not (Figure 5k). These findings confirmed that HB52 binds directly to the *PUT2* promoter and may regulate *PUT2* expression in response to salt stress.

### 2.5 Disruption of PUT2 Abolishes the Salt Tolerance of *HB52*‐OE Plants

**Figure 6:**
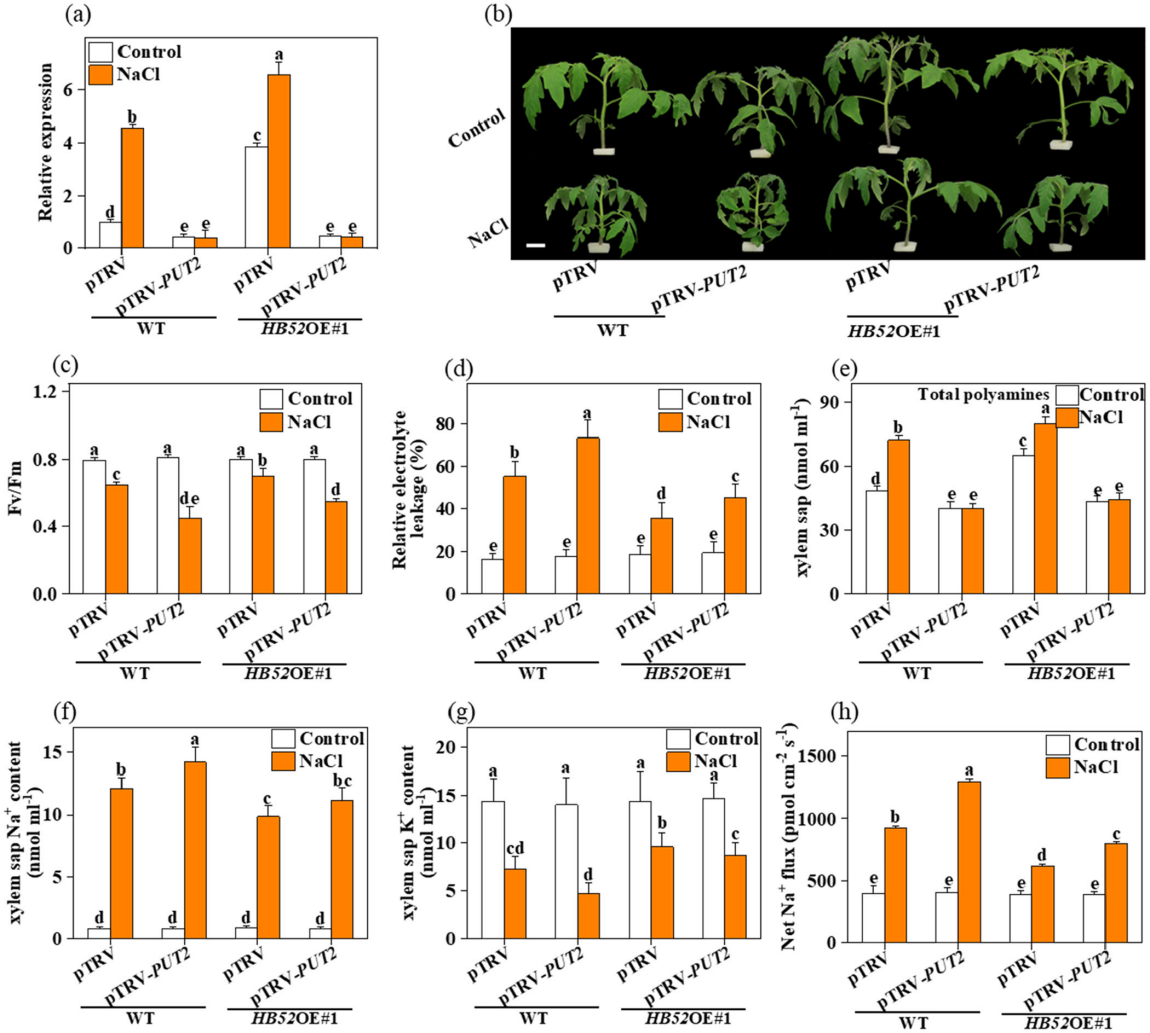
Functional analysis of PUT2 in HB52‐induced salt tolerance. (a) The expression levels of *PUT2* in silenced lines under salt conditions, *actin* was used as the internal control. (b) Phenotypes for the silencing of *PUT2* in WT or *HB52*OE plants exposed salt treatment for 7 days, Bar = 5 cm; (c, d) The Fv/Fm and REL in WT, *hb52* mutant, and *HB52*‐OE plants were exposed to salt treatment for 7 days; (e) The total polyamine content in xylem sap in silenced lines exposed to salt treatment for 7 days; (f–h) The Na^+^, K^+^, and Na^+^/K^+^ ratio in xylem sap in silenced lines exposed to salt treatment for 7 days; Data are presented as mean values ± SD; *n* = 3. Different letters indicate significant treatment differences (*p* < 0.05, Duncan's multiple range test). At least three independent experiments were performed. [Color figure can be viewed at wileyonlinelibrary.com]

To further confirm the role of PUT2 in HB52‐regulated shoot‐to‐root polyamine transport, we silenced the *PUT2* gene in WT and *HB52*‐OE lines using a tobacco rattle virus (TRV) virus‐induced gene silencing (VIGS) system. The *PUT2* transcript levels were reduced by 60% to 85% in gene‐silenced plants than in the TRV control plants, and the phytoene phenotype was observed in the TRV‐phytoene desaturase plants (Figure S11). The expression of *PUT2* was induced under salt stress but was significantly decreased in the TRV‐*PUT2* and *HB52*‐OE TRV‐*PUT2* plantlets compared to that of the TRV control (Figure 6a). Importantly, the PUT2‐silenced WT and *HB52*‐OE plants exhibited a distinct reduction in salt tolerance (Figure 6b).

In addition, *PUT2* silencing abolished the observed induction of *F*_*v*_ */F*_*m*_ and the decline of REL in the *HB52*‐OE background under salt stress (Figure 6c,d). Salt treatment increased the levels of polyamines in the xylem sap by 49.6% in the WT and by 65.8% in *HB52*‐OE plantlets; silencing of *PUT2* compromised this salt‐induced increase of polyamines in the xylem sap (Figure 6e). The Na^+^ content decreased, and the K^+^ content increased significantly in the xylem sap in the *HB52*‐OE seedlings under salt stress, whereas the plants with silenced *PUT2* accumulated more Na^+^ and less K^+^ in the xylem sap than those of the TRV control plants (Figure 6f,g); the same trend was found for the Na^+^ efflux capacity of the roots (Figure 6h). Collectively, these results indicated that HB52‐regulated salt tolerance is largely dependent on the PUT2‐mediated polyamine shoot‐to‐root mobility.

## 3 Discussion

Plant polyamine transport fulfills a series of functions, but is primarily associated with eliciting various physiological responses to stress. A rising consensus has emphasised the close relationship between polyamine transport and stress responses, particularly in the responses to heat stress (Shen et al. 2016). However, the roles of polyamine transport and the molecular mechanisms involved in the response to salinity stress are largely known. In this study, we demonstrated that under salt stress, the shoot‐to‐root polyamine translocation mediated by PUT2 leads to a decreased xylem sap Na^+^ content, and the HB52‐PUT2 interaction increases shoot‐to‐root polyamine mobility, thereby attenuating root‐to‐shoot Na^+^ translocation, ultimately conferring salt tolerance in tomato.

To date, the majority of research on the coordination of shoot and root processes regulated by polyamines has focused on the changes in polyamine biosynthesis that occur in plants in response to stress. Evidence suggests that the accumulation of polyamines responsible for morphological changes in the root under stress are biosynthesized directly in the root. In contrast to this concept, exogenous application of putrescine to the leaves was reported to induce drastic fluctuations in endogenous polyamine levels, subsequently affecting growth and development (Bagni, Baraldi and Costa 1983). Recent studies have helped to clarify the molecular factors contributing to polyamine transport in eukaryotic cells and those that stringently regulate polyamine levels in cells (Li et al. 2013). Biosynthesis of polyamines in cells represents the main source of polyamines in plants kingdom; however, the cellular synthesis of polyamines in response to various environmental cues is a time‐ and energy‐consuming process. When a rapid elevation in polyamine content is required under stress conditions, polyamine pools in the extracellular spaces serve as a faithful and readily accessible bank for inducing an intracellular increase of polyamine through regulating their short‐ or long‐distance transport rather than their *de novo* synthesis. However, the mechanisms by which polyamine synthesised in the shoots is transported to the roots in response to salt stress have not been elucidated until now.

In this study, we found that the *adc1* mutant displayed higher salt sensitivity and a lower polyamine content than those of the WT after salt treatment, which suggested that the decreased salt tolerance of this mutant is at least partly due to the low concentration of polyamine (Figure 1). Polyamines are translocated within the plant from the shoots to the down parts and vice versa (Caffaro et al. 1993). A basipetal transport of radioactivity was observed after feeding labelled polyamines to cotyledons or mature leaves (Beraud et al. 1992). Furthermore, our findings support that the changed polyamine levels under salt stress are largely driven by shoot‐to‐root transport. In particular, salt treatment increased the content of polyamine in the leaves, roots, and xylem sap of WT plants, but did not induce the accumulation of polyamine in the tissues of *adc1* mutant plants; however, foliar putrescine treatment increased the accumulation of polyamine in the leaves, roots, and xylem sap of both WT and *adc1* mutant plants (Figure 1). Therefore, we concluded that there exists a pathway regulating the transport of polyamine from the shoots to the roots, and this process plays a vital role in enhancing salt tolerance.

Recently, plant transporters involved in polyamine transport have gained increasing attention. PUT proteins are conserved l‐type amino acid transporters and essential regulators of polyamine in different organelles and cellular signal transduction (Abdulhussein and Wallace 2014; Do et al. 2019). PUT2 is a member of the PUT family that has been shown to play versatile roles in plant development and the response to stress in *Arabidopsis* (Dong et al. 2016; Kim et al. 2019). Our previous physiological study indicated that PUT2 enhances salt tolerance by regulating the polyamine pathway (Zhong et al. 2023). Recent studies have demonstrated that manipulation of polyamine‐related genes leads to plant dwarfism. The gain‐of‐function mutant *wox1*‐D (WUSCHEL HOMEOBOX 1) showed significantly lesser polyamine contents and SAMDC activity, resulting in the dwarf phenotype (Zhang et al. 2011). In the *acl5* (T‐Spm synthase) knockout mutant of *Arabidopsi*s, the mutant showed severe dwarfism due to the excessive proliferation and differentiation of the xylem (Takano et al. 2012). Indeed, knockout of *put2* reduced shoot growth under normal conditions (Figure 2a). Here, we found that overexpression of tomato PUT2 positively regulated Na^+^ efflux and inhibited Na^+^ transport from the roots to shoots (Figure 2). This result was consistent with a previous study showing that the *Arabidopsis put3* mutant exhibited Na^+^ accumulation or K^+^ deficiency, causing an ionic imbalance in cells exposed to salt stress (Chai et al. 2020). The same research group also found that PUT3 could interact with the Na^+^/H^+^ antiporters SOS1 and SOS2, subsequently forming a complex to alter the activity of PUT3 and SOS1 in response to environmental cues (Chai et al. 2020). The main function of SOS is to prevent Na^+^ from entering the leaves by expelling Na^+^ from the xylem parenchyma cells (Zhu et al. 2016). In addition, the *Arabidopsis* homologue of PUT2 has also been associated with polyamine transport activity and oxidative damage counteraction in *Arabidopsis* (Fujita and Shinozaki 2014). Thus, control of Na^+^ efflux is essential for PUT2‐induced salt tolerance under salt stress.

Polyamine synthesis in vivo is distributed in various organs, and the polyamine is then extensively inter‐converted across tissues. Nonetheless, the polyamine content is carefully controlled to maintain cellular homoeostasis in various cellular activities via PUTs, and PUTs are involved in polyamine transport across the plasma membrane and intracellular translocation, fine‐tuning optimal organellar polyamine pool in the cells (Strohm et al. 2015). Shoot‐to‐root transport is a crucial part of this process, which was further highlighted in this study based on the essential role of PUT2 in the distribution of polyamine. On the one hand, the essential role of PUT2 in the shoot‐to‐root long‐distance movement of polyamine is backed by the partial recovery of salt tolerance in the *put2* mutant upon grafting of a WT scion. On the other hand, the failure to recover salt tolerance upon grafting a *put2* mutant (scion) onto the WT (rootstock) corroborated the pivotal role of PUT2 in this process (Figure 3). Moreover, PUT2 was implicated in the shoot‐to‐root translocation of polyamine based on the total and root polyamine content reduction when using the *put2* mutant as the rootstock plant (Figure 3). Further analysis of individual polyamines displayed that spermidine and putrescine are the main polyamines in shoot‐to‐root transport, and their levels were significantly reduced in the *put2* mutant. Thus, PUT2 may prefer spermidine and putrescine shoot‐to‐root mobility, which can then be converted to other polyamines or hydrogen peroxide by the corresponding metabolic synthases, activating suitable defence mechanisms to enhance resistance against salt stress.

Although the transcriptional regulation of polyamine synthesis and metabolism genes has been reported (Fujita et al. 2012; Li et al. 2022; Song et al. 2023), it has remained unclear whether there are special TFs that regulate PUT genes, particularly *PUT2*. HD‐Zip proteins have a broad array of functions governing a series of hormone signalling pathways and abiotic and biotic stress responses (Ariel et al. 2007). This study found that *HB52* expression was rapidly induced by salt treatment, hormones, and polyamines (Figure 4a,b, Figure 5a, and Figure S10). This indicated that this HD‐Zip protein may be crucial in salt stress resistance via the polyamine transport pathway. Indeed, knockout of *HB52* markedly impaired the tolerance of tomato plants to salt stress, while the overexpression lines displayed the opposite phenotypes with respect to *F*_*v*_ */F*_*m*_ and REL (Figure 4e). Lower levels of Na^+^ and a higher K^+^ content were detected in the xylem vessels of *HB52*‐OE plants than those of WT plants when exposed to salt stress, whereas *hb52* mutants showed a significantly different pattern (Figure 4f–h), indicating that alteration of Na^+^ unloading from the xylem vessels may play an important role in HB52‐regulated salt tolerance. Furthermore, the levels of total polyamine decreased in the xylem sap of the *hb52* mutants. They increased in *HB52*‐OE plants compared to WT, with or without salt treatment, suggesting that *HB52* is involved in the long‐distance transport of polyamine, potentially interacting with *PUT2* (Figure 4i). In contrast, in the WT xylem saps, spermidine and putrescine were the prevalent polyamines, but the levels of these polyamines were strongly decreased in *hb52* and increased in *HB52*‐OE plants (Figure 4j). The surprising increase in total polyamine content in the xylem vessels of *HB52*‐OE plants, thereby contributing to polyamine accumulation in the roots, can provide a molecular explanation for the increased salt tolerance. Root polyamines have been suggested to play an essential role in enhanced salinity tolerance by improving the capacity of Na^+^ extrusion and Na^+^/K^+^ homoeostasis (Zarza et al. 2020). HB52 is involved in a wide range of hormone signalling, including ethylene and auxin (Miao et al. 2018). The interplay of polyamines and hormones has been widely reported. For example, polyamines interplay with IAA, ABA, and ethylene pathways to regulate fruit ripening, seed generation, and leaf development (Yang et al. 2024). Therefore, the role of polyamines in coordinating HB52 and hormones in regulating stress responses needs to be further investigated.

Strikingly, the *PUT2* expression level was reduced in *hb52* mutants but increased in *HB52*‐OE lines (Figure 5b), indicating that HB52 may be an upstream regulator for *PUT2*. In *Arabidopsis*, HB52 binds to the promoter of VAR2 to improve light energy utilisation under low nitrogen conditions; HB52 also regulates auxin transport genes such as PIN2 and WAG1 (Miao et al. 2018; Ariga et al. 2022). Here, our in vitro and in vivo binding experiments (Figure 5) displayed that HB52 could physically bind to the promoter region of *PUT2*. Based on the LUC assay results, HB52 can activate *PUT2* transcripts but has different effects on mutant *PUT2* promoter‐dependent transcription (Figure 5c,g). In addition, our findings demonstrate that *PUT2* is a direct target of *HB52* in HB52‐mediated salt tolerance. Our ChIP‐qPCR and yeast experiment results showed that in the *PUT2* promoter, only two putative HD binding sites were bound by HB52 (Figure 5). Similarly, the sequence‐specific binding of HB52 was confirmed by LUC reporter assays and EMSAs (Figure 5), indicating that the other putative binding sites may not be functional or may be bound by other homeodomain proteins. In addition, *PUT2* silencing in *HB52*‐OE plants resulted in hypersensitivity to salt stress and completely abolished HB52‐induced plant salt tolerance (Figure 6a–c). Meanwhile, the induced shoot‐to‐root transport of polyamine in *HB52*‐OE plants was strongly reduced by silencing *PUT2* (Figure 6d). The *HB52*‐OE *PUT2*‐silenced plants further exhibited increased Na^+^ and decreased K^+^ in xylem parenchyma cells (Figure 6e,f). Thus, HB52 directly regulates the expression of *PUT2* to enhance salt tolerance via the PUT2‐mediated polyamine shoot‐to‐root mobility.

## 4 Conclusion

**Figure 7:**
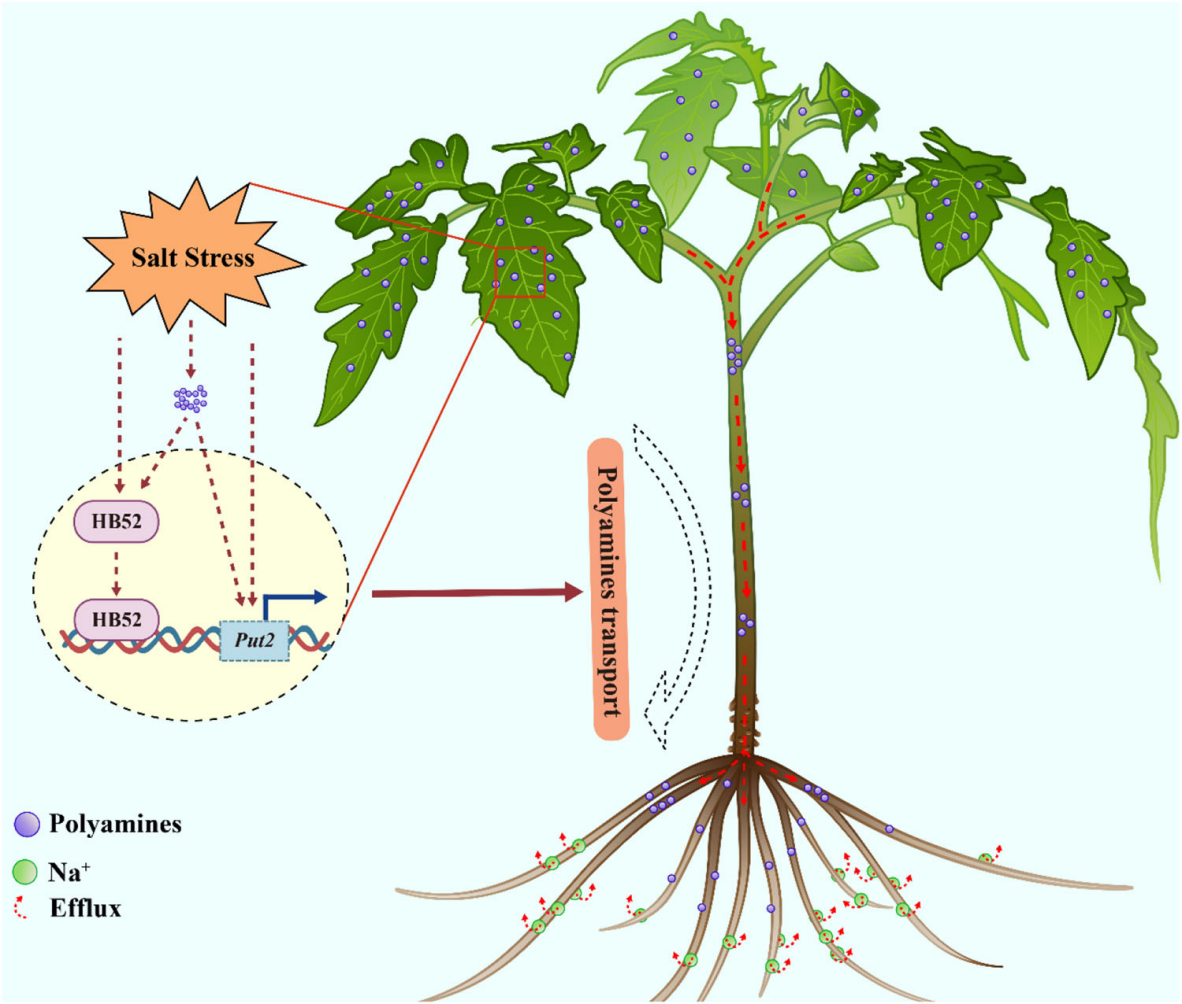
Working model depicting the function of HB52 in salt stress. Transcription factor HB52 promotes the shoot‐to‐root polyamines translocation by inducing the expression of the PUT2. Polyamine positively regulates HB52 expression, forming a positive loop to enhance salt tolerance. [Color figure can be viewed at wileyonlinelibrary.com]

In summary, our findings demonstrate that the salt‐induced HB52‐PUT2 cascade plays an important role in the adaptation of plants to salt stress by induction of the long‐distance transport of polyamine. The salt‐induced accumulation of HB52 directly activates the expression of *PUT2*. PUT2, which in turn enhances the salt tolerance of plants by directly increasing polyamine shoot‐to‐root translocation. In addition, PUT2 itself also enhances salt tolerance independent of HB52 (Figure 7). Our results thus expand current knowledge of the roles of PUT2 and shed new light on the mechanisms of the long‐distance transport of polyamine in the response to salt stress. Importantly, this study reveals the HB52‐PUT2 module as a vital component in regulating polyamine mobility, and enhancing salt tolerance.

## 5 Experimental Procedures

### 5.1 Plant Materials and Growth Conditions

The wild‐type (WT) tomato (*Solanum lycopersicum* L. cv Ailsa Craig), and mutant plants (one CRISPR‐Cas 9 *adc* mutant line, two CRISPR‐Cas 9 *put2*#1 and *put2*#2 mutant lines and two CRISPR‐Cas 9 *hb52#1* and *hb52#2* mutant lines) and overexpression transgenic lines (*HB52*‐OE#1 and *HB52*‐OE#2) in the same genetic background (Ailsa Craig) were used in this study. The seedlings were grown in a hydroponic tank with Hoagland's nutrient solution, and placed in growth chambers with a 12‐h/12‐h light/dark photoperiod at 600 μmol m^−^^2^ s^−^^1^. *Nicotiana benthamiana* was grown at 25°C/20°C (day/night) with a 16‐h/8‐h light/dark photoperiod for transient expression.

### 5.2 Construction of Transgenic Plants

To generate loss‐function of lines, the target sequences for *adc1*, *put2* and *hb52* were designed using the online software CRISPR‐GE (http://skl.scau.edu.cn/targetdesign/), respectively. Then, these sequences were inserted into guide RNA (sgRNA) expression cassettes through overlap PCR, followed by cloning into the pYLCRISPR/Cas9Pubi‐H vector via the Golden Gate ligation method (Xie et al. 2017). Transgenic plants were then generated using the Agrobacterium‐mediated method of cotyledon transformation of *Solanum lycopersicum* cv. Ailsa Craig. To identify *adc1*, *put2*, and *hb52* mutant plants, we analyzed the mutation by the PCR amplification and Sanger sequencing; homozygotes in T1 progeny plants were used for further study, the information on *put2* mutant has been previously described (Zhong et al. 2023). To obtain the *HB52‐OE* tomato lines, full‐length *HB52* cDNA was amplified by PCR and cloned into the pFGC1008‐HA vector, followed by transformation in the same manner as used for the mutant constructs. The primers are listed in Table S1.

TRV VIGS constructs were used to silence the target gene (*PUT2*) with the TRV‐based vectors (pTRV1/2). The target gene fragments were PCR‐amplified and inserted into the pTRV2 vector; the empty vector of pTRV2 served as a control. The sequencing vector was transformed into *Agrobacterium tumefaciens* strain GV3101, and virus infection was performed as previously described (Liu, Schiff and Dinesh‐Kumar 2002). Plants were grown in a growth chamber at 22°C for 4 weeks and then used for experiments; the gene silencing efficiency was evaluated by RT‐qPCR (Livak and Schmittgen 2001). The primers used for the VIGS experiments are listed in Table S1.

### 5.3 Salt Treatment and Tolerance Assays

Tomato plants at four‐leaf stages were used for salt treatment. The tomato seedlings were randomly divided into two groups, and the seedlings in Group 1 were transferred to the hydroponic system with 0 mM NaCl as a Control. The other group was grown in full Hoagland's nutrient solution with 125‐mM NaCl. All seedlings were grown with a new nutrient solution every 3 days and intermittently aerated, with a 12‐h photoperiod. White LED luminaires provided supporting illumination to maintain a total light level of approximately 600 μmol m^−^^2^ s^−^^1^ in the plant canopy. Daytime and nighttime temperatures ragged 24 ± 2°C/20 ± 2°C, respectively, with humidity maintained at 70%–80%. For salt tolerance, photographs of plants were captured after being treated for 7 days under salt treatment. The REL was measured based on electric conductivity, and the Fv/Fm was determined by a Dual‐PAM‐100 system (Walz, Germany), as previously described (Zhong et al. 2023). For the treatment with putrescine, the WT and *adc1* mutant plants were sprayed with eight mM putrescine (Sigma‐Aldrich, 51799) every 2 days, and the control plants were sprayed with the same amount of distilled H_2_O.

### 5.4 Polyamine Transport Assays

For the PUT2‐mediated polyamine transport assay, 2‐week‐old seedlings were placed in 1 L of 1/2 Hoagland nutrient solution. After 12 h incubation, foliar sprays of two µM ^13^C‐spermidine (Sigma‐Aldrich, 740780), three replicates were performed. The roots of the seedlings were cut off after 3 h of treatment, washed three times, and transferred into a 2 mL centrifuge tube. The radioactive measurement was used at a Beckman LS‐6500 scintillation counter (Beckman coulter, USA). The transport rate was determined according to the amount of spermidine per hour per gram of protein and fresh weight (Kang et al. 2024; Shen et al. 2016).

### 5.5 Grafting Experiment

To investigate the role of PUT2 in the shoots and roots, one salt tolerance and polyamine translocation, hypocotyl‐to‐hypocotyl grafts were established using a wedge grafting technique. Shoots of WT and *put2* seedlings at the three‐leaf stage were self‐grafted or reciprocally grafted onto the rootstocks of WT and *put2*, respectively, resulting in WT/WT, WT/*put2*, *put2/*WT, and *put2/put2* lines of grafted plants. A small plastic clip and specialised tape for grafting were used to hold the scion in place. For the first 3 days, grafted plants were placed in sealed, light‐transmitting plastic containers to maintain high humidity, which promoted graft wounding healing. In addition, slow or weak grafts were discarded so that all graft combinations represented robust individuals. Plants with the same number of nodes were selected for further analysis.

### 5.6 Collection of Xylem Sap

Tomato plants or graft seedlings were grown hydroponically in Hoagland's nutrient solution for 4 weeks in a grow chamber under 16‐/8‐ light/dark photoperiod at 600 μmol m^−^^2^ s^−1^ at 24 ± 2°C/20 ± 2°C (day/night), and then normal growth or salt stress treatment. The plants were then kept in a chamber with 70‐80% humidity, and their stems were cut horizontally. The water droplets on the cut surface of the stem were collected (Lu et al. 2023; Zhong et al. 2023).

### 5.7 Determinant of Na^+^ and K^+^ Content

The levels of Na^+^ and K^+^ were determined according to the method of Zhong et al. (2023), with some modifications. The shoots and roots of the plants were collected after 7 days of salt treatment, and the samples were placed in an oven at 75°C for 2 days to obtain the permanent dry weight.

### 5.8 Determinant of Free Polyamines Content

Free polyamines were measured by HPLC (Agilent Technologies 1200) as described by Zhong et al. (2023) with slight modifications. Samples were harvested and homogenised in 5% (v/v) cold trichloroacetic acid (TCA) and incubated at four °C for one h, followed by centrifugation for 20 min at 12 000 × *g*; the supernatant was quantitatively transferred into another vial. A 500 μL of 2 M NaOH was added, followed by 2.5 μL 50% (v/v) TCA, mixed well, and incubated at 25°C for 40 min. Then, 500 μL of saturated was added to terminate the reaction, and benzoylated polyamines were extracted with 700 μL of diethyl ether. Ether was evaporated to dryness, redissolved in 100 μL of 65% (v/v) methanol, and stored at ‐80°C until analysis. Polyamines were measured using an HPLC 1200 series system with a C18 column. Samples were eluted from the column with 65% methanol at a flow rate of 0.8 mL min^‐1^. Standards of polyamines and other chemicals and reagents were purchased from Sigma Aldrich chemical company (St. Louis, MO, USA).

### 5.9 Electrophoretic Mobility Shift Assay

The full‐length coding sequence of HB52 was inserted into the pGEX‐4T‐1 vector, and this expression box was converted into *Escherichia coli* strain BL21 (DE3). The protein of GST‐HB52 was induced by 0.1 mM isopropyl β‐D‐1‐thiogalactopyranoside, and was purified by Glutathione‐Superflow Resin (Takara). For EMSA, the probes were designed and labelled with biotin, which was annealed to obtain double‐stranded probes. A Light Shift chemiluminescent EMSA kit (Thermo Scientific, 20148) was used to detect the biotin‐labelled DNA according to the manufacturer's instructions. The primers are listed in Table S1.

### 5.10 Reverse Transcription‐Quantitative Polymerase Chain Reaction

To analyze the expression of salinity tolerance‐related genes, plant total RNA was isolated by an RNA extraction reagent (Tiangen, DP419) and reverse‐transcribed with the HiScriptTM qRT SuperMix for qPCR (+gDNA wiper) kit (Vazyme, #R323). Real‐time PCR was then performed on an ABI VII7 real‐time PCR system (Applied Biosystems, Waltham, MA, USA), with each reaction containing ten ng first‐strand cDNA 100 ng, ten mmol L^‐1^ gene‐specific primers, and 12.5 μL of real‐time ChamQ SYBR Color qPCR Master Mix (Vazyme, Q421‐02). The thermal cycling programme consisted of 95°C for 5 min, followed by 40 cycles of 95°C for 5 s, and then 60°C for 1 min. The tomato actin gene was used as the endogenous reference. The primers used for RT‐qPCR are listed in Table S1.

### 5.11 Yeast One‐Hybrid (Y1H) Assay

Y1H assay experiments were performed as described for the Gold Yeast One‐Hybrid kit (Takara Bio Inc., USA). The target DNA sequence *proPUT2* (approximately 2 kb of the PUT2 promoters) was inserted into the vector pAbAi, and then recombined into the genome of the Y1H Gold‐yeast strain. The yeast one‐hybrid (Y1H) assay was performed from a tomato cDNA library using the full‐length PUT2 promoter as bait. Positive clones were obtained and sequenced, and HB52 was found. The HB52 full‐length cDNA was amplified and cloned into the pGADT7 prey vector. To get the mutant, the sequence CAAT(A/T)ATTG was replaced by CAGAGGTTG using the Mut Express MultiS Fast Mutagenesis Kit V2 (Vazyme C215‐01) according to the manufacturer's instructions. The yeast strain Y1H Gold was transfected with the HB52 protein vector (pGADT7‐HB52) and the linearised promoter vector (pAbAi‐_pro_PUT2). The transformed yeast cells were identified on selection plates (SD/Leu‐medium+100 ng mL^−^^1^ aureobasidin A). The primers for yeast one‐hybrid amplification are listed in Table S1.

### 5.12 ChIP‐qPCR Assay

The *HB52*‐OE line was used for ChIP experiments with the EpiQuik Plant ChIP Kit (Epigentek P‐2014). Approximately ~2 g of the leaf was sampled from WT and HB52‐OE plants. The tissues were cross‐linked in 1% formaldehyde by vacuuming for 10 min, and then 0.1 M Gly was added to stop cross‐linking. The sample was ground in liquid nitrogen and used to isolate nuclei; the chromatin was sonicated to obtain 200–500 bp fragments. HA‐antibody (Pierce 26183) was used to immunoprecipitate the protein‐DNA complex, and the precipitated DNA was recovered and analyzed by ChIP‐qPCR. Chromatin precipitated with goat anti‐mouse IgG (Millipore AP124P) was used as the negative control. Primers used for ChIP‐qPCR are listed in Table S1.

### 5.13 Dual‐Luciferase Assay

The full‐length coding sequence of *HB52* was inserted into the pGreen II 62‐SK vector, and the amplified fragment of the *PUT2* promoter was cloned into the pGreen II 0800‐SK vector. The effector and reporter were transiently transferred into tobacco leaves for 48 h for co‐expression. The LUC activity was measured 2 days later with the Dual‐Luciferase reporter assay kit (Promega) and normalised to *Renilla* activity. The empty vector pGreen II 62‐SK is driven by the 35S promoter as the control. The primers used for establishing these constructs are listed in Table S1.

### 5.14 Root Na^+^ Flux Analysis

An NMT kit (Xuyue Sci. & Tech. Co. Ltd., Beijing, China) was used to measure the net Na^+^ flux. In brief, seedlings were fixed in measuring solution (0.5 mM NaCl, 0.2 mM Na_2_SO_4_, 0.3 mM MES, 0.1 mM KCl, 0.1 mM CaCl_2_, 0.1 mM MgCl_2_; pH = 6) under a Na^+^ ion‐selective microelectrode, and the probe was propelled into the root elongation zone. Net Na^+^ efflux monitoring in the root apex at a distance of 0 ~ 500 μm was used to validate the maximum Na^+^ fluctuation region. The flux of Na^+^ was recorded for at least 5 min. More than three individual seedlings were used for measurements under each point.

### 5.15 Xylem Parenchyma Cell Na^+^ Flux Analysis

To assess Na^+^ flux in xylem parenchyma cells, 1‐month‐old tomato seedlings were treated with 120 mM NaCl for 24 h. The primary root was cut from the root‐rhizome junction and then fully balanced in measuring solution (the solution used in the root Na^+^ flux analysis) for over 20 min. Samples were fixed in Petri dishes using resin blocks with filter paper containing the measuring solution. The micro‐electrode was positioned before the parenchyma cell to measure the Na^+^ flux.

## Conflicts of Interest

The authors declare no conflicts of interest.

## Supporting information

Supporting information.

Supporting information.

## Data Availability

The authors confirm that the data supporting the findings of this study are available within the article and its supporting information.

## References

[pce15479-bib-1101] Abdulhussein, A. A. , and H. M. Wallace . 2014. “Polyamines and Membrane Transporters.” Amino Acids 46: 655–660. 23851697 10.1007/s00726-013-1553-6

[pce15479-bib-0001] Alhag, A. , J. Song , B. Dahro , et al. 2021. “Genome‐Wide Identification and Expression Analysis of Polyamine Uptake Transporter Gene Family in Sweet Orange (*Citrus sinensis*).” Plant Biology 23, no. 6: 1157–1166. 34374185 10.1111/plb.13302

[pce15479-bib-0002] Ariel, F. D. , P. A. Manavella , C. A. Dezar , and R. L. Chan . 2007. “The True Story of the HD‐Zip Family.” Trends in Plant Science 12, no. 9: 419–426. 17698401 10.1016/j.tplants.2007.08.003

[pce15479-bib-0003] Ariga, T. , Y. Sakuraba , M. Zhuo , M. Yang , and S. Yanagisawa . 2022. “The Arabidopsis NLP7‐HB52/54‐VAR2 Pathway Modulates Energy Utilization in Diverse Light and Nitrogen Conditions.” Current Biology 32, no. 24: 5344–5353.e5346. 36332616 10.1016/j.cub.2022.10.024

[pce15479-bib-0004] Bagni, N. , R. Baraldi , and G. Costa . 1983. “Uptake, Translocation and Metabolism of Aliphatic Polyamines in Leaves and Fruitlets of Malus domestica (cv. Ruby Spur).” Flowering and Fruit Set in Fruit Trees 149: 173–178.

[pce15479-bib-0005] Beraud, J. , A. Brun , A. Feray , A. Hourmant , and M. Penot . 1992. “Long Distance Transport of ^14^C‐putrescine in Potato Plantlets (*Solanum tuberosum* cv. Bintje).” Biochemie und Physiologie der Pflanzen 188, no. 3: 169–176.

[pce15479-bib-1001] Caffaro, S. , S. Scaramagli , F. Antognoni , and N. Bagni . 1993. “Polyamine Content and Translocation in Soybean Plants.” Journal of Plant Physiology 141, no. 5: 563–568.

[pce15479-bib-0007] Chai, H. , J. Guo , Y. Zhong , et al. 2020. “The Plasma‐Membrane Polyamine Transporter PUT3 Is Regulated by the Na+/H+ Antiporter SOS1 and Protein Kinase Sos2.” New Phytologist 226, no. 3: 785–797. 31901205 10.1111/nph.16407

[pce15479-bib-0008] Chen, D. , Q. Shao , L. Yin , A. Younis , and B. Zheng . 2019. “Polyamine Function in Plants: Metabolism, Regulation on Development, and Roles in Abiotic Stress Responses.” Frontiers in Plant Science 9: 1945. 30687350 10.3389/fpls.2018.01945 PMC6335389

[pce15479-bib-0009] Do, T. H. T. , H. Choi , M. Palmgren , E. Martinoia , J.‐U. Hwang , and Y. Lee . 2019. “Arabidopsis ABCG28 Is Required for the Apical Accumulation of Reactive Oxygen Species in Growing Pollen Tubes.” Proceedings of the National Academy of Sciences 116, no. 25: 12540–12549. 10.1073/pnas.1902010116 PMC6589667 31152136

[pce15479-bib-0010] Dong, S. , H. Hu , Y. Wang , et al. 2016. “A pqr2 Mutant Encodes a Defective Polyamine Transporter and Is Negatively Affected by ABA for Paraquat Resistance in *Arabidopsis thaliana* .” Journal of Plant Research 129: 899–907. 27229891 10.1007/s10265-016-0819-y

[pce15479-bib-0011] Fujita, M. , Y. Fujita , S. Iuchi , et al. 2012. “Natural Variation in a Polyamine Transporter Determines Paraquat Tolerance in Arabidopsis.” Proceedings of the National Academy of Sciences 109, no. 16: 6343–6347. 10.1073/pnas.1121406109 PMC3341036 22492932

[pce15479-bib-0012] Fujita, M. , and K. Shinozaki . 2014. “Identification of Polyamine Transporters in Plants: Paraquat Transport Provides Crucial Clues.” Plant and Cell Physiology 55, no. 5: 855–861. 24590488 10.1093/pcp/pcu032

[pce15479-bib-0013] Gémes, K. , Y. J. Kim , K. Y. Park , et al. 2016. “An NADPH‐Oxidase/Polyamine Oxidase Feedback Loop Controls Oxidative Burst Under Salinity.” Plant Physiology 172, no. 3: 1418–1431. 27600815 10.1104/pp.16.01118 PMC5100782

[pce15479-bib-0014] Gerlin, L. , C. Baroukh , and S. Genin . 2021. “Polyamines: Double Agents in Disease and Plant Immunity.” Trends in Plant Science 26, no. 10: 1061–1071. 34127368 10.1016/j.tplants.2021.05.007

[pce15479-bib-0015] Gong, X. , J. Zhang , J. Hu , et al. 2015. “Fcwrky 70, a WRKY Protein of F Ortunella Crassifolia, Functions in Drought Tolerance and Modulates Putrescine Synthesis by Regulating Arginine Decarboxylase Gene.” Plant, Cell & Environment 38, no. 11: 2248–2262. 10.1111/pce.12539 25808564

[pce15479-bib-0016] González‐Hernández, A. I. , L. Scalschi , P. Troncho , P. García‐Agustín , and G. Camañes . 2022. “Putrescine Biosynthetic Pathways Modulate Root Growth Differently in Tomato Seedlings Grown Under Different N Sources.” Journal of Plant Physiology 268: 153560. 34798464 10.1016/j.jplph.2021.153560

[pce15479-bib-1003] Guo, M. , X.‐S. Wang , H.‐D. Guo , et al. 2022. “Tomato Salt Tolerance Mechanisms and Their Potential Applications for Fighting Salinity: A Review.” Frontiers in Plant Science 13: 949541. 36186008 10.3389/fpls.2022.949541 PMC9515470

[pce15479-bib-0017] Ismail, A. M. , and T. Horie . 2017. “Genomics, Physiology, and Molecular Breeding Approaches for Improving Salt Tolerance.” Annual Review of Plant Biology 68: 405–434. 10.1146/annurev-arplant-042916-040936 28226230

[pce15479-bib-0018] Jiang, J. , S. Ma , N. Ye , M. Jiang , J. Cao , and J. Zhang . 2017. “WRKY Transcription Factors in Plant Responses to Stresses.” Journal of Integrative Plant Biology 59, no. 2: 86–101. 27995748 10.1111/jipb.12513

[pce15479-bib-0019] Joshi, R. , S. H. Wani , B. Singh , et al. 2016. “Transcription Factors and Plants Response to Drought Stress: Current Understanding and Future Directions.” Frontiers in Plant Science 7: 204078. 10.3389/fpls.2016.01029 PMC4943945 27471513

[pce15479-bib-0020] Kang, Y. , H. Qin , G. Wang , B. Lei , X. Yang , and M. Zhong . 2024. “Selenium Nanoparticles Mitigate Cadmium Stress in Tomato through Enhanced Accumulation and Transport of Sulfate/Selenite and Polyamines.” Journal of Agricultural and Food Chemistry 72, no. 3: 1473–1486. 38214288 10.1021/acs.jafc.3c07504 PMC10811693

[pce15479-bib-0021] Kim, W. , S. Ć. Zeljković , U. Piskurewicz , C. Megies , P. Tarkowski , and L. Lopez‐Molina . 2019. “Polyamine Uptake Transporter 2 (put2) and Decaying Seeds Enhance phyA‐Mediated Germination by Overcoming PIF1 Repression of Germination.” PLoS Genetics 15, no. 7: e1008292. 31339933 10.1371/journal.pgen.1008292 PMC6682160

[pce15479-bib-0022] Kundu, A. , S. Mishra , P. Kundu , A. Jogawat , and J. Vadassery . 2022. “Piriformospora Indica Recruits Host‐Derived Putrescine for Growth Promotion in Plants.” Plant Physiology 188, no. 4: 2289–2307. 34791442 10.1093/plphys/kiab536 PMC8968253

[pce15479-bib-1004] Lan Thi Hoang, X. , N. H. Du Nhi , N. Binh Anh Thu , N. Phuong Thao , and L.‐S. Phan Tran . 2017. “Transcription Factors and Their Roles in Signal Transduction in Plants Under Abiotic Stresses.” Current Genomics 18, no. 6: 483–497. 29204078 10.2174/1389202918666170227150057 PMC5684650

[pce15479-bib-1005] Li, J. , J. Mu , J. Bai , et al. 2013. “Paraquat Resistant1, a Golgi‐Localized Putative Transporter Protein, Is Involved in Intracellular Transport of Paraquat.” Plant Physiology 162, no. 1: 470–483. 23471133 10.1104/pp.113.213892 PMC3641224

[pce15479-bib-0026] Li, M. , X. Duan , G. Gao , T. Liu , and H. Qi . 2022. “CmABF1 and CmCBF4 Cooperatively Regulate Putrescine Synthesis to Improve Cold Tolerance of Melon Seedlings.” Horticulture Research 9: uhac002.35147169

[pce15479-bib-0027] Liu, J.‐H. , W. Wang , H. Wu , X. Gong , and T. Moriguchi . 2015. “Polyamines Function in Stress Tolerance: From Synthesis to Regulation.” Frontiers in Plant Science 6: 827. 26528300 10.3389/fpls.2015.00827 PMC4602114

[pce15479-bib-0028] Liu, T. , J. Qu , Y. Fang , et al. 2024. “Polyamines: The Valuable Bio‐Stimulants and Endogenous Signaling Molecules for Plant Development and Stress Response.” Journal of Integrative Plant Biology: 1–14. 10.1111/jipb.13796 39601632

[pce15479-bib-0029] Liu, X. , S. Liu , X. Chen , et al. 2022. “Maize miR167‐ARF3/30‐polyamine Oxidase 1 Module‐Regulated H2O2 Production Confers Resistance to Maize Chlorotic Mottle Virus.” Plant Physiology 189, no. 2: 1065–1082. 35298645 10.1093/plphys/kiac099 PMC9157100

[pce15479-bib-0030] Liu, Y. , M. Schiff , and S. P. Dinesh‐Kumar . 2002. “Virus‐Induced Gene Silencing in Tomato.” Plant Journal 31, no. 6: 777–786. 10.1046/j.1365-313x.2002.01394.x 12220268

[pce15479-bib-0032] Livak, K. J. , and T. D. Schmittgen . 2001. “Analysis of Relative Gene Expression Data Using Real‐Time Quantitative PCR and the 2− ΔΔCT Method.” Methods 25, no. 4: 402–408. 11846609 10.1006/meth.2001.1262

[pce15479-bib-0033] Lu, K.‐K. , R.‐F. Song , J.‐X. Guo , et al. 2023. “CycC1; 1–WRKY75 Complex‐Mediated Transcriptional Regulation of SOS1 Controls Salt Stress Tolerance in Arabidopsis.” Plant Cell 35, no. 7: 2570–2591. 37040621 10.1093/plcell/koad105 PMC10291036

[pce15479-bib-1002] Lyu, Y.‐S. , L.‐M. Cao , W.‐Q. Huang , J.‐X. Liu , and H.‐P. Lu . 2022. “Disruption of Three Polyamine Uptake Transporter Genes in Rice by CRISPR/Cas9 Gene Editing Confers Tolerance to Herbicide Paraquat.” aBIOTECH 3, no. 2: 140–145. 36304519 10.1007/s42994-022-00075-4 PMC9590464

[pce15479-bib-0036] Miao, Z.‐Q. , P.‐X. Zhao , J.‐L. Mao , et al. 2018. “HOMEOBOX PROTEIN52 Mediates the Crosstalk Between Ethylene and Auxin Signaling During Primary Root Elongation by Modulating Auxin Transport‐Related Gene Expression.” Plant Cell 30, no. 11: 2761–2778. 30333147 10.1105/tpc.18.00584 PMC6305987

[pce15479-bib-0037] Milhinhos, A. , J. Prestele , B. Bollhöner , et al. 2013. “Thermospermine Levels Are Controlled by an Auxin‐Dependent Feedback Loop Mechanism in Populus Xylem.” Plant Journal 75, no. 4: 685–698. 10.1111/tpj.12231 23647338

[pce15479-bib-0038] Min, D. , J. Zhou , J. Li , et al. 2021. “SlMYC2 Targeted Regulation of Polyamines Biosynthesis Contributes to Methyl Jasmonate‐Induced Chilling Tolerance in Tomato Fruit.” Postharvest Biology and Technology 174: 111443.

[pce15479-bib-0039] Mulangi, V. , M. C. Chibucos , V. Phuntumart , and P. F. Morris . 2012. “Kinetic and Phylogenetic Analysis of Plant Polyamine Uptake Transporters.” Planta 236: 1261–1273. 22711282 10.1007/s00425-012-1668-0

[pce15479-bib-0040] Mulangi, V. , V. Phuntumart , M. Aouida , D. Ramotar , and P. Morris . 2012. “Functional Analysis of OsPUT1, a Rice Polyamine Uptake Transporter.” Planta 235: 1–11. 21796369 10.1007/s00425-011-1486-9

[pce15479-bib-0041] Pál, M. , G. Szalai , O. K. Gondor , and T. Janda . 2021. “Unfinished Story of Polyamines: Role of Conjugation, Transport and Light‐Related Regulation in the Polyamine Metabolism in Plants.” Plant Science 308: 110923. 34034871 10.1016/j.plantsci.2021.110923

[pce15479-bib-0042] Pál, M. , G. Szalai , and T. Janda . 2015. “Speculation: Polyamines Are Important in Abiotic Stress Signaling.” Plant Science 237: 16–23. 26089148 10.1016/j.plantsci.2015.05.003

[pce15479-bib-0043] Ré, D. A. , M. Capella , G. Bonaventure , and R. L. Chan . 2014. “Arabidopsis AtHB7 and AtHB12 Evolved Divergently to Fine Tune Processes Associated With Growth and Responses to Water Stress.” BMC Plant Biology 14: 150. 24884528 10.1186/1471-2229-14-150 PMC4064807

[pce15479-bib-0044] Sen, S. , J. Chakraborty , P. Ghosh , D. Basu , and S. Das . 2017. “Chickpea WRKY70 Regulates the Expression of a Homeodomain‐Leucine Zipper (HD‐Zip) I Transcription Factor CaHDZ12, Which Confers Abiotic Stress Tolerance in Transgenic Tobacco and Chickpea.” Plant and Cell Physiology 58, no. 11: 1934–1952. 29016956 10.1093/pcp/pcx126

[pce15479-bib-0045] Shen, Y. , Q. Ruan , H. Chai , et al. 2016. “The Arabidopsis Polyamine Transporter LHR 1/PUT 3 Modulates Heat Responsive Gene Expression by Enhancing Mrna Stability.” Plant Journal 88, no. 6: 1006–1021. 10.1111/tpj.13310 27541077

[pce15479-bib-0046] Song, J. , P. Sun , W. Kong , Z. Xie , C. Li , and J. H. Liu . 2023. “SnRK2. 4‐mediated Phosphorylation of ABF2 Regulates Arginine Decarboxylase Expression and Putrescine Accumulation Under Drought Stress.” New Phytologist 238, no. 1: 216–236. 36210523 10.1111/nph.18526

[pce15479-bib-0047] Stolarska, E. , U. K. Tanwar , Y. Guan , et al. 2023. “Genetic Portrait of Polyamine Transporters in Barley: Insights in the Regulation of Leaf Senescence.” Frontiers in Plant Science 14: 1194737. 37332717 10.3389/fpls.2023.1194737 PMC10272464

[pce15479-bib-0048] Strohm, A. K. , L. M. Vaughn , and P. H. Masson . 2015. “Natural Variation in the Expression of Organic Cation Transporter 1 Affects Root Length Responses to Cadaverine in Arabidopsis.” Journal of Experimental Botany 66, no. 3: 853–862. 25403917 10.1093/jxb/eru444 PMC4321547

[pce15479-bib-0049] Takano, A. , J.‐I. Kakehi , and T. Takahashi . 2012. “Thermospermine Is not a Minor Polyamine in the Plant Kingdom.” Plant and Cell Physiology 53, no. 4: 606–616. 22366038 10.1093/pcp/pcs019

[pce15479-bib-0050] Thongbhubate, K. , Y. Nakafuji , R. Matsuoka , S. Kakegawa , and H. Suzuki . 2021. “Effect of Spermidine on Biofilm Formation in *Escherichia coli* K‐12.” Journal of Bacteriology 203, no. 10: e00652‐20. 33685971 10.1128/JB.00652-20 PMC8088603

[pce15479-bib-0051] Van Veen, S. , S. Martin , C. Van den Haute , et al. 2020. “ATP13A2 Deficiency Disrupts Lysosomal Polyamine Export.” Nature 578, no. 7795: 419–424. 31996848 10.1038/s41586-020-1968-7

[pce15479-bib-0052] Vrijsen, S. , L. Besora‐Casals , S. van Veen , et al. 2020. “ATP13A2‐Mediated Endo‐Lysosomal Polyamine Export Counters Mitochondrial Oxidative Stress.” Proceedings of the National Academy of Sciences 117, no. 49: 31198–31207. 10.1073/pnas.1922342117 PMC7733819 33229544

[pce15479-bib-0053] Wu, D. , E. von Roepenack‐Lahaye , M. Buntru , et al. 2019. “A Plant Pathogen Type III Effector Protein Subverts Translational Regulation to Boost Host Polyamine Levels.” Cell Host & Microbe 26, no. 5: 638–649. 31628081 10.1016/j.chom.2019.09.014

[pce15479-bib-0054] Xie, X. , X. Ma , Q. Zhu , D. Zeng , G. Li , and Y.‐G. Liu . 2017. “CRISPR‐GE: A Convenient Software Toolkit for CRISPR‐Based Genome Editing.” Molecular Plant 10, no. 9: 1246–1249. 28624544 10.1016/j.molp.2017.06.004

[pce15479-bib-0055] Yang, H. , Y. Fang , Z. Liang , T. Qin , J. H. Liu , and T. Liu . 2024. “Polyamines: Pleiotropic Molecules Regulating Plant Development and Enhancing Crop Yield and Quality.” Plant Biotechnology Journal 22, no. 11: 3194–3201. 39024414 10.1111/pbi.14440 PMC11500986

[pce15479-bib-0056] Yin, L. , S. Wang , K. Tanaka , et al. 2016. “Silicon‐Mediated Changes in Polyamines Participate in Silicon‐Induced Salt Tolerance in S Orghum Bicolor L.” Plant, Cell & Environment 39, no. 2: 245–258. 10.1111/pce.12521 25753986

[pce15479-bib-0057] Zarza, X. , R. Van Wijk , L. Shabala , et al. 2020. “Lipid Kinases PIP5K7 and PIP5K9 Are Required for Polyamine‐Triggered K+ Efflux in Arabidopsis Roots.” Plant Journal 104, no. 2: 416–432. 10.1111/tpj.14932 PMC7693229 32666545

[pce15479-bib-0058] Van Zelm, E. , Y. Zhang , and C. Testerink . 2020. “Salt Tolerance Mechanisms of Plants.” Annual Review of Plant Biology 71: 403–433. 10.1146/annurev-arplant-050718-100005 32167791

[pce15479-bib-0060] Zhang, J. Z. 2003. “Overexpression Analysis of Plant Transcription Factors.” Current Opinion in Plant Biology 6, no. 5: 430–440. 12972043 10.1016/s1369-5266(03)00081-5

[pce15479-bib-0061] Zhang, Y. , R. Wu , G. Qin , Z. Chen , H. Gu , and L. J. Qu . 2011. “Over‐Expression of WOX1 Leads to Defects in Meristem Development and Polyamine Homeostasis in Arabidopsis F.” Journal of Integrative Plant Biology 53, no. 6: 493–506. 21658178 10.1111/j.1744-7909.2011.01054.x

[pce15479-bib-0062] Zhong, M. , L. Yue , W. Liu , et al. 2023. “Genome‐Wide Identification and Characterization of the Polyamine Uptake Transporter (Put) Gene Family in Tomatoes and the Role of Put2 in Response to Salt Stress.” Antioxidants 12, no. 2: 228. 36829787 10.3390/antiox12020228 PMC9952195

[pce15479-bib-0063] Zhong, M. , L. Yue , H. Qin , et al. 2023. “TGase‐Induced CD Tolerance by Boosting Polyamine, Nitric Oxide, Cell Wall Composition and Phytochelatin Synthesis in Tomato.” Ecotoxicology and Environmental Safety 259: 115023. 37201425 10.1016/j.ecoenv.2023.115023

[pce15479-bib-0064] Zhu, J.‐K. 2002. “Salt and Drought Stress Signal Transduction in Plants.” Annual Review of Plant Biology 53, no. 1: 247–273. 10.1146/annurev.arplant.53.091401.143329 PMC3128348 12221975

[pce15479-bib-0065] Zhu, M. , L. Shabala , T. A. Cuin , et al. 2016. “Nax Loci Affect SOS1‐Like Na^+^/H^+^ Exchanger Expression and Activity in Wheat.” Journal of Experimental Botany 67, no. 3: 835–844. 26585227 10.1093/jxb/erv493 PMC4737075

