# Supplementary material for: HB52‐PUT2 Module‐Mediated Polyamine Shoot‐to‐Root Movement Regulates Salt Stress Tolerance in Tomato
Source: Plant Cell Environ. 2025 Mar 30;48(7):5148–63. doi: 10.1111/pce.15479 (PMC12131959; doi:10.1111/pce.15479)
Supplement: Supplementary file 2 — Supporting information. [file PCE-48-5148-s001.docx]

**SUPPORTING INFORMATION**

**Additional Supporting Information may be found in the online version of this article.**

**Figure S1**. **Generation of *adc1* mutant by CRISPR-Cas9 system. The black horizontal line indicates sgRNA.** The red font indicates protospacer-adjacent motif (PAM) sequences, and the black dash indicates deletion. The *adc1* line has a one bp deletion, leading to a premature termination of the ADC protein.

**Figure S2**. **The Fv/Fm (a) and REL (b) in WT and *adc1* mutant after exogenous putrescine treatment under salt stress.**

Data are presented as mean values±SD; n=3. Different letters indicate signiﬁcant treatment differences (*P*<0.05, Duncan’s multiple range test). At least three independent experiments were performed.

**Figure S3**. **The REL in WT, *put2* mutant, and PUT2-OE plants were exposed to salt treatment for seven days.**

Data are presented as mean values±SD; n=3. Different letters indicate signiﬁcant treatment differences (*P*<0.05, Duncan’s multiple range test). At least three independent experiments were performed.

**Figure S4**. **The** **spermidine (a), putrescine (b), spermine (c), and total polyamines (d) content in WT, *put2* mutant, and PUT2-OE plants were exposed to salt treatment for seven days.**

Data are presented as mean values±SD; n=3. Different letters indicate signiﬁcant treatment differences (*P*<0.05, Duncan’s multiple range test). At least three independent experiments were performed.

**Figure S5**. **Spermidine uptake assays in the *put2* mutant and WT plants (a). The Fv/Fm in grafted plants with *put2* as rootstock or scion exposed salt treatment for seven days (b).**

Data are presented as mean values±SD; n=3. Different letters indicate signiﬁcant treatment differences (*P*<0.05, Duncan’s multiple range test). At least three independent experiments were performed.

**Figure S6**. **The** **spermine (Spm) accumulation in leaf (a) and root (b) in grafted plants with *put2* as rootstock or scion.**

Data are presented as mean values±SD; n=3. Different letters indicate signiﬁcant treatment differences (*P*<0.05, Duncan’s multiple range test). At least three independent experiments were performed.

**Figure S7**. **Identification of *HB52* overexpression lines by qRT-PCR.**

Data are presented as mean values±SD; n=3. Different letters indicate signiﬁcant treatment differences (*P*<0.05, Duncan’s multiple range test). At least three independent experiments were performed.

**Figure S8**. **The REL in WT, *hb52* mutant, and *HB52*-OE plants were exposed to salt treatment for seven days.**

Data are presented as mean values±SD; n=3. Different letters indicate signiﬁcant treatment differences (*P*<0.05, Duncan’s multiple range test). At least three independent experiments were performed.

**Figure S9**. **The ion content in the shoots and roots of different genotypes.**

Plots showing Na^+^ (a, c) and K^+^ (b, d) contents of shoots or roots of 4-week-old seedlings of the indicated genotypes treated with or without 125 mM NaCl for 5 d (n=5 plants per genotype), respectively.

Data are presented as mean values±SD; n=3. Different letters indicate signiﬁcant treatment differences (*P*<0.05, Duncan’s multiple range test). At least three independent experiments were performed. DW, dry weight.

**Figure S10**. **Expression pattern of *HB52* and response to abiotic stresses.**

(a) qRT-PCR results show the expression of HB52 in different plant tissues, including stem, root, seed, leaf, and flower. (b and c) The transcript level of HB52 in response to different phytohormones. 3-week-old wild-type seedlings were separated with 10 MeJA or ten μM ABA for 0, 0.5, 1, 2, 3, 6, 12, 24 h.

The experiment was repeated three times. Data are means ± SD (n = 3). *Actin* was used as a reference gene. Different letters indicate signiﬁcant treatment differences (*P*<0.05, Duncan’s multiple range test). At least three independent experiments were performed.

**Figure S11**. **Phenotypes for the silencing of *PDS* in WT background, Bar=5 cm.**

**Table S1. List of primers used in this study.**

**Figure legend**

**Figure 1**. **Effect of long-distance polyamine transport on the phenotype of WT and putrescine biosynthetic mutants (*adc1*) of tomato under salt stress.**

(a) Phenotypes of exogenous putrescine-mediated salt tolerance in WT and *adc1* mutant, photographs were taken seven days after treatment, Bar=5 cm;

(b, c, and d) Total polyamines content in leaf (b), root (c), and xylem sap (d) in WT and *adc1* mutant after exogenous putrescine treatment under salt stress for seven days.

Data are presented as mean values±SD; n=3. Different letters indicate signiﬁcant treatment differences (*P*<0.05, Duncan’s multiple range test). At least three independent experiments were performed.

**Figure 2**. **Polyamine uptake protein 2 (PUT2) positively regulates salt tolerance.**

(a) Phenotypes of WT, *put2* mutant and *PUT2*-overexpression (*PUT2*-OE) plants exposed salt treatment for 7 days, Bar=5 cm;

(b) The Fv/Fm in WT, *put2* mutant, and *PUT2*-OE plants exposed to salt treatment for seven days;

(c) Net Na^+^ fluxes in 500 μm distance from root apex of WT, *put2* mutant, and *PUT2*-OE plants under salt treatment;

(d) Net Na^+^ fluxes of the root xylem parenchyma cell in the roots of WT, *put2* mutant, and *PUT2*-OE plants under the treatment of 120 mM NaCl for 24 h;

(e) Quantitative analysis of the means of net Na^+^ fluxes within a continuous period of 0 to 5 min.

Data are presented as mean values±SD; n=3. Different letters indicate signiﬁcant treatment differences (*P*<0.05, Duncan’s multiple range test). At least three independent experiments were performed.

**Figure 3. PUT2-dependent shoot-to-root polyamine transport is essential for salt resistance.**

(a) Phenotypes in grafted plants with *put2* as rootstock or scion with or without salt treatment for seven days;

(b) The Na^+^ content in root in grafted plants with *put2* as rootstock or scion exposed salt treatment for seven days;

(c, e, and g) Total polyamines, spermidine (Spd), and putrescine (Put) accumulation in leaf in grafted plants with *put2* as rootstock or scion with or without salt treatment for seven days;

(d, f and h) Total polyamines, Spd, and Put accumulation in root in grafted plants with *put2* as rootstock or scion with or without salt treatment for seven days;

Data are presented as mean values±SD; n=3. Different letters indicate signiﬁcant treatment differences (*P*<0.05, Duncan’s multiple range test). At least three independent experiments were performed.

**Figure 4**. **HB52 positively regulates salt tolerance.**

(a, b) Time course analysis of putrescine-induced *HB52* expression of leaf (a) and root (b) in WT seedlings;

(c) Generation of *hb52* mutants by CRISPR-Cas9 system. Blue letters indicate protospacer-adjacent motif (PAM) sequences and red dash indicates deletion; the *hb52*#1 line has a seven bp deletion, and the *hb52*#2 line has a five bp deletion, leading to early termination of HB52 protein, respectively;

(d) Phenotypes of WT, *hb52* mutant and *HB52*-overexpression (*HB52*-OE) plants exposed salt treatment for 7 days; Bar=5 cm;

(e) The Fv/Fm in WT, *hb52* mutant, and *HB52*-OE plants exposed to salt treatment for seven days;

(f, g and h) The Na^+^, K^+^ and Na^+^/K^+^ ratio in xylem sap in WT, *hb52* mutant and *HB52*-OE plants exposed salt treatment for 7 days;

(i) The total polyamines content in xylem sap in WT, *hb52* mutant, and *HB52*-OE plants exposed to salt treatment for seven days;

(j) The individual polyamines putrescine, spermidine, and spermine levels of the same samples are shown in j.

Data are presented as mean values±SD; n=3. Different letters indicate signiﬁcant treatment differences (*P*<0.05, Duncan’s multiple range test). At least three independent experiments were performed.

**Figure 5. HB52 positively regulates salt tolerance by activating *PUT2* expression**

(a) Relative expression of *PUT2* in leaves and roots. Three-week-old seedlings were treated with or without 120 NaCl for 48 h;

(b) Expression levels of *PUT2* in three-week-old WT, *put2* mutans, and *PUT2*-OE plants;

(c) Transient expression of HB52 in tobacco leaves activates *PUT2* promoter activity; (d) Schematic diagram of *PUT2* promoter. Vertical blue bars indicate putative HB52-binding sites. Grey horizontal lines represent the PCR fragments (F1 to F4) for the ChIP assays in f. Black horizontal lines represent the probes (P1 to P3) for the Y1H assays in h;

(e) Reporter constructs for co-transfection assays. Red lowercase letters represent nucleotide substitutions introduced into the consensus sequence to generate mutated *PUT2* promoter and mutant EMSA probe;

(f) ChIP-qPCR assays indicate that HB52 binds to the *PUT2* promoter in vivo. The data show relative enrichment of DNA precipitated with Flag antibody to those treated with IgG (set to 1). The fragments in an exon of *PUT2* and promoter of Ubi were used as controls. Data are means ± SD (n = 3);

(g) Dual-luciferase assay for the regulatory effect of HB52 on the expression of *PUT2*. WT and mutated *PUT2* promoter were used for the assay. The ratio of LUC/REN of the empty plus promoter was set as one;

(h) Yeast one-hybrid (Y1H) experiment showing the binding of HB52-AD to the P2 or P3 regions of the *PUT2* promoter;

(i) Y1H experiment shows HB52 could not bind to the mutated P2 or P3 regions of the *PUT2* promoter.

(j) Dual-luciferase assay shows HB52 could not bind to the mutated P2P3 regions of the *PUT2* promoter;

(k) EMSA assay. The HB52:GST recombinant protein was incubated with biotin-labeled WT (PUT2-P2-wt, PUT2-P3-wt) or mutant (PUT2-P2-m, PUT2-P3-m) oligos. The protein purified from the empty vector was used as a negative control. In (B), data represent the mean ± SD of three biological replicates. Asterisks (***p*<0.01; Student’s t-test) indicate significant differences identified between WT plants and other samples (f), between samples using HB52 expression and empty plasmids (g).

**Figure 6. Functional analysis of PUT2 in HB52-induced salt tolerance.**

(a) The expression levels of *PUT2* in silenced lines under salt conditions, *actin* was used as the internal control.

(b) Phenotypes for the silencing of *PUT2* in WT or *HB52*OE plants exposed salt treatment for 7 days, Bar=5 cm;

(c, d) The Fv/Fm and REL in WT, *hb52* mutant, and *HB52*-OE plants were exposed to salt treatment for seven days;

(e) The total polyamine content in xylem sap in silenced lines exposed to salt treatment for seven days;

(f, g and h) The Na^+^, K^+^, and Na^+^/ K^+^ ratio in xylem sap in silenced lines exposed to salt treatment for seven days;

Data are presented as mean values±SD; n=3. Different letters indicate signiﬁcant treatment differences (*P*<0.05, Duncan’s multiple range test). At least three independent experiments were performed.

**Figure 7. Working model depicting the function of HB52 in salt stress.** Transcription factor HB52 promotes the shoot-to-root polyamines translocation by inducing the expression of the PUT2. Polyamine positively regulates HB52 expression, forming a positive loop to enhance salt tolerance.

**Reference**

Alhag, A., Song, J., Dahro, B., Wu, H., Khan, M., Salih, H., & Liu, J. H. (2021). Genome‐wide identification and expression analysis of Polyamine Uptake Transporter gene family in sweet orange (*Citrus sinensis*). *Plant Biology, 23*(6), 1157-1166.

Ariel, F. D., Manavella, P. A., Dezar, C. A., & Chan, R. L. (2007). The true story of the HD-Zip family. *Trends in Plant Science, 12*(9), 419-426.

Ariga, T., Sakuraba, Y., Zhuo, M., Yang, M., & Yanagisawa, S. (2022). The Arabidopsis NLP7-HB52/54-VAR2 pathway modulates energy utilization in diverse light and nitrogen conditions. *Current Biology, 32*(24), 5344-5353. e5346.

Bagni, N., Baraldi, R., & Costa, G. (1983). Uptake, translocation and metabolism of aliphatic polyamines in leaves and fruitlets of Malus domestica (cv. Ruby Spur). *Flowering and Fruit Set in Fruit Trees 149*, 173-178.

Beraud, J., Brun, A., Feray, A., Hourmant, A., & Penot, M. (1992). Long distance transport of ^14^C-putrescine in potato plantlets (Solanum tuberosum cv. Bintje). *Biochemie Und Physiologie Der Pflanzen*, 188(3), 169-176.

Caffaro, S., Scaramagli, S., Antognoni, F., & Bagni, N. (1993). Polyamine content and translocation in soybean plants. *Journal of plant physiology*, 141(5), 563-568.

Chai, H., Guo, J., Zhong, Y., Hsu, C. C., Zou, C., Wang, P., . . . Shi, H. (2020). The plasma‐membrane polyamine transporter PUT3 is regulated by the Na+/H+ antiporter SOS1 and protein kinase SOS2. *New phytologist, 226*(3), 785-797.

Chen, D., Shao, Q., Yin, L., Younis, A., & Zheng, B. (2019). Polyamine function in plants: metabolism, regulation on development, and roles in abiotic stress responses. *Frontiers in Plant Science, 9*, 1945.

Do, T. H. T., Choi, H., Palmgren, M., Martinoia, E., Hwang, J.-U., & Lee, Y. (2019). Arabidopsis ABCG28 is required for the apical accumulation of reactive oxygen species in growing pollen tubes. *Proceedings of the National Academy of Sciences, 116*(25), 12540-12549.

Dong, S., Hu, H., Wang, Y., Xu, Z., Zha, Y., Cai, X., . . . Feng, S. (2016). A pqr2 mutant encodes a defective polyamine transporter and is negatively affected by ABA for paraquat resistance in Arabidopsis thaliana. *Journal of plant research, 129*, 899-907.

Fujita, M., Fujita, Y., Iuchi, S., Yamada, K., Kobayashi, Y., Urano, K., . . . Shinozaki, K. (2012). Natural variation in a polyamine transporter determines paraquat tolerance in Arabidopsis. *Proceedings of the National Academy of Sciences, 109*(16), 6343-6347.

Fujita, M., & Shinozaki, K. (2014). Identification of polyamine transporters in plants: paraquat transport provides crucial clues. *Plant and cell physiology, 55*(5), 855-861.

Gémes, K., Kim, Y. J., Park, K. Y., Moschou, P. N., Andronis, E., Valassaki, C., . . . Roubelakis-Angelakis, K. A. (2016). An NADPH-oxidase/polyamine oxidase feedback loop controls oxidative burst under salinity. *Plant physiology, 172*(3), 1418-1431.

Gerlin, L., Baroukh, C., & Genin, S. (2021). Polyamines: double agents in disease and plant immunity. *Trends in Plant Science, 26*(10), 1061-1071.

Gong, X., Zhang, J., Hu, J., Wang, W., Wu, H., Zhang, Q., & Liu, J. H. (2015). FcWRKY 70, a WRKY protein of F ortunella crassifolia, functions in drought tolerance and modulates putrescine synthesis by regulating arginine decarboxylase gene. *Plant, Cell & Environment, 38*(11), 2248-2262.

González-Hernández, A. I., Scalschi, L., Troncho, P., García-Agustín, P., & Camañes, G. (2022). Putrescine biosynthetic pathways modulate root growth differently in tomato seedlings grown under different N sources. Journal of plant physiology, 268, 153560.

Ismail, A. M., & Horie, T. (2017). Genomics, physiology, and molecular breeding approaches for improving salt tolerance. *Annual review of plant biology, 68*, 405-434.

Jiang, J., Ma, S., Ye, N., Jiang, M., Cao, J., & Zhang, J. (2017). WRKY transcription factors in plant responses to stresses. *Journal of integrative plant biology, 59*(2), 86-101.

Joshi, R., Wani, S. H., Singh, B., Bohra, A., Dar, Z. A., Lone, A. A., . . . Singla-Pareek, S. L. (2016). Transcription factors and plants response to drought stress: current understanding and future directions. *Frontiers in Plant Science, 7*, 204078.

Kang, Y., Qin, H., Wang, G., Lei, B., Yang, X., & Zhong, M. (2024). Selenium Nanoparticles Mitigate Cadmium Stress in Tomato through Enhanced Accumulation and Transport of Sulfate/Selenite and Polyamines. *Journal of Agricultural and Food Chemistry, 72*(3), 1473-1486.

Kim, W., Zeljković, S. Ć., Piskurewicz, U., Megies, C., Tarkowski, P., & Lopez-Molina, L. (2019). Polyamine uptake transporter 2 (put2) and decaying seeds enhance phyA-mediated germination by overcoming PIF1 repression of germination. *PLoS genetics, 15*(7), e1008292.

Kundu, A., Mishra, S., Kundu, P., Jogawat, A., & Vadassery, J. (2022). Piriformospora indica recruits host-derived putrescine for growth promotion in plants. Plant physiology, 188(4), 2289-2307.

Lan Thi Hoang, X., Du Nhi, N. H., Binh Anh Thu, N., Phuong Thao, N., & Phan Tran, L.-S. (2017). Transcription factors and their roles in signal transduction in plants under abiotic stresses. *Current genomics, 18*(6), 483-497.

Li, J., Li, Q., Guo, N., Xian, Q., Lan, B., Nangia, V., . . . Liu, Y. (2024). Polyamines mediate the inhibitory effect of drought stress on nitrogen reallocation and utilization to regulate grain number in wheat. *Journal of Experimental Botany, 75*(3), 1016-1035.

Li, J., Mu, J., Bai, J., Fu, F., Zou, T., An, F., . . . Li, Z. (2013). Paraquat Resistant1, a Golgi-localized putative transporter protein, is involved in intracellular transport of paraquat. *Plant physiology, 162*(1), 470-483.

Li, M., Duan, X., Gao, G., Liu, T., & Qi, H. (2022). CmABF1 and CmCBF4 cooperatively regulate putrescine synthesis to improve cold tolerance of melon seedlings. *Horticulture Research, 9*, uhac002.

Liu, J.-H., Wang, W., Wu, H., Gong, X., & Moriguchi, T. (2015). Polyamines function in stress tolerance: from synthesis to regulation. Frontiers in Plant Science, 6, 827.

Liu, T., Qu, J., Fang, Y., Yang, H., Lai, W., Pan, L., & Liu, J. H. (2024). Polyamines: The valuable bio‐stimulants and endogenous signaling molecules for plant development and stress response. *Journal of integrative plant biology*.

Liu, X., Liu, S., Chen, X., Prasanna, B. M., Ni, Z., Li, X., . . . Zhou, T. (2022). Maize miR167-ARF3/30-polyamine oxidase 1 module-regulated H2O2 production confers resistance to maize chlorotic mottle virus. *Plant physiology, 189*(2), 1065-1082.

Liu, Y., Schiff, M., & Dinesh‐Kumar, S. P. (2002). Virus‐induced gene silencing in tomato. *The Plant Journal, 31*(6), 777-786.

Livak, K. J., & Schmittgen, T. D. (2001). Analysis of relative gene expression data using real-time quantitative PCR and the 2− ΔΔCT method. *methods, 25*(4), 402-408.

Lu, K.-K., Song, R.-F., Guo, J.-X., Zhang, Y., Zuo, J.-X., Chen, H.-H., . . . Lu, Y.-T. (2023). CycC1; 1–WRKY75 complex-mediated transcriptional regulation of SOS1 controls salt stress tolerance in Arabidopsis. *The Plant Cell, 35*(7), 2570-2591.

Lyu, Y.-S., Cao, L.-M., Huang, W.-Q., Liu, J.-X., & Lu, H.-P. (2022). Disruption of three polyamine uptake transporter genes in rice by CRISPR/Cas9 gene editing confers tolerance to herbicide paraquat. *Abiotech, 3*(2), 140-145.

Martinis, J., Gas-Pascual, E., Szydlowski, N., Crèvecoeur, M., Gisler, A., Bürkle, L., & Fitzpatrick, T. B. (2016). Long-distance transport of thiamine (vitamin B1) is concomitant with that of polyamines. *Plant physiology, 171*(1), 542-553.

Miao, Z.-Q., Zhao, P.-X., Mao, J.-L., Yu, L.-H., Yuan, Y., Tang, H., . . . Xiang, C.-B. (2018). HOMEOBOX PROTEIN52 mediates the crosstalk between ethylene and auxin signaling during primary root elongation by modulating auxin transport-related gene expression. *The Plant Cell, 30*(11), 2761-2778.

Milhinhos, A., Prestele, J., Bollhöner, B., Matos, A., Vera‐Sirera, F., Rambla, J. L., . . . Tuominen, H. (2013). Thermospermine levels are controlled by an auxin‐dependent feedback loop mechanism in Populus xylem. *The Plant Journal, 75*(4), 685-698.

Min, D., Zhou, J., Li, J., Ai, W., Li, Z., Zhang, X., . . . Li, X. (2021). SlMYC2 targeted regulation of polyamines biosynthesis contributes to methyl jasmonate-induced chilling tolerance in tomato fruit. *Postharvest Biology and Technology, 174*, 111443.

Mulangi, V., Chibucos, M. C., Phuntumart, V., & Morris, P. F. (2012). Kinetic and phylogenetic analysis of plant polyamine uptake transporters. *Planta, 236*, 1261-1273.

Mulangi, V., Phuntumart, V., Aouida, M., Ramotar, D., & Morris, P. (2012). Functional analysis of OsPUT1, a rice polyamine uptake transporter. *Planta, 235*, 1-11.

Pál, M., Szalai, G., Gondor, O. K., & Janda, T. (2021). Unfinished story of polyamines: Role of conjugation, transport and light-related regulation in the polyamine metabolism in plants. *Plant Science, 308*, 110923.

Pál, M., Szalai, G., & Janda, T. (2015). Speculation: polyamines are important in abiotic stress signaling. *Plant Science, 237*, 16-23.

Ré, D. A., Capella, M., Bonaventure, G., & Chan, R. L. (2014). Arabidopsis AtHB7 and AtHB12 evolved divergently to fine tune processes associated with growth and responses to water stress. *BMC plant biology, 14*, 1-14.

Sen, S., Chakraborty, J., Ghosh, P., Basu, D., & Das, S. (2017). Chickpea WRKY70 regulates the expression of a homeodomain-leucine zipper (HD-Zip) I transcription factor CaHDZ12, which confers abiotic stress tolerance in transgenic tobacco and chickpea. *Plant and cell physiology, 58*(11), 1934-1952.

Shen, Y., Ruan, Q., Chai, H., Yuan, Y., Yang, W., Chen, J., . . . Shi, H. (2016). The Arabidopsis polyamine transporter LHR 1/PUT 3 modulates heat responsive gene expression by enhancing mRNA stability. *The Plant Journal, 88*(6), 1006-1021.

Song, J., Sun, P., Kong, W., Xie, Z., Li, C., & Liu, J. H. (2023). SnRK2. 4‐mediated phosphorylation of ABF2 regulates ARGININE DECARBOXYLASE expression and putrescine accumulation under drought stress. *New phytologist, 238*(1), 216-236.

Stolarska, E., Tanwar, U. K., Guan, Y., Grabsztunowicz, M., Arasimowicz-Jelonek, M., Phanstiel IV, O., & Sobieszczuk-Nowicka, E. (2023). Genetic portrait of polyamine transporters in barley: insights in the regulation of leaf senescence. *Frontiers in Plant Science, 14*, 1194737.

Strohm, A. K., Vaughn, L. M., & Masson, P. H. (2015). Natural variation in the expression of ORGANIC CATION TRANSPORTER 1 affects root length responses to cadaverine in Arabidopsis. *Journal of Experimental Botany, 66*(3), 853-862.

Takano, A., Kakehi, J.-I., & Takahashi, T. (2012). Thermospermine is not a minor polyamine in the plant kingdom. *Plant and cell physiology*, 53(4), 606-616.

Thongbhubate, K., Nakafuji, Y., Matsuoka, R., Kakegawa, S., & Suzuki, H. (2021). Effect of spermidine on biofilm formation in Escherichia coli K-12. *Journal of Bacteriology, 203*(10), 10.1128/jb. 00652-00620.

Van Veen, S., Martin, S., Van den Haute, C., Benoy, V., Lyons, J., Vanhoutte, R., . . . Lambie, E. (2020). ATP13A2 deficiency disrupts lysosomal polyamine export. Nature, 578(7795), 419-424.

Van Zelm, E., Zhang, Y., & Testerink, C. (2020). Salt tolerance mechanisms of plants. *Annual review of plant biology, 71*, 403-433.

Vrijsen, S., Besora-Casals, L., van Veen, S., Zielich, J., Van den Haute, C., Hamouda, N. N., . . . Agostinis, P. (2020). ATP13A2-mediated endo-lysosomal polyamine export counters mitochondrial oxidative stress. *Proceedings of the National Academy of Sciences, 117*(49), 31198-31207.

Wu, D., von Roepenack-Lahaye, E., Buntru, M., de Lange, O., Schandry, N., Pérez-Quintero, A. L., . . . Michael, A. J. (2019). A plant pathogen type III effector protein subverts translational regulation to boost host polyamine levels. Cell Host & Microbe, 26(5), 638-649. e635.

Xie, X., Ma, X., Zhu, Q., Zeng, D., Li, G., & Liu, Y.-G. (2017). CRISPR-GE: a convenient software toolkit for CRISPR-based genome editing. *Molecular plant, 10*(9), 1246-1249.

Yang, H., Fang, Y., Liang, Z., Qin, T., Liu, J. H., & Liu, T. (2024). Polyamines: pleiotropic molecules regulating plant development and enhancing crop yield and quality. *Plant Biotechnology Journal*. 22(11), 3194-3201.

Yang, L., Hong, X., WEN, X.-X., & LIAO, Y.-C. (2016). Effect of polyamine on seed germination of wheat under drought stress is related to changes in hormones and carbohydrates. *Journal of Integrative Agriculture, 15*(12), 2759-2774.

Yin, L., Wang, S., Tanaka, K., Fujihara, S., Itai, A., Den, X., & Zhang, S. (2016). Silicon‐mediated changes in polyamines participate in silicon‐induced salt tolerance in S orghum bicolor L. *Plant, Cell & Environment, 39*(2), 245-258.

Zarza, X., Van Wijk, R., Shabala, L., Hunkeler, A., Lefebvre, M., Rodriguez‐Villalón, A., . . . Munnik, T. (2020). Lipid kinases PIP5K7 and PIP5K9 are required for polyamine‐triggered K+ efflux in Arabidopsis roots. *The Plant Journal, 104*(2), 416-432.

Zhang, H., Zhu, J., Gong, Z., & Zhu, J.-K. (2022). Abiotic stress responses in plants. *Nature Reviews Genetics, 23*(2), 104-119.

Zhang, J. Z. (2003). Overexpression analysis of plant transcription factors. *Current opinion in plant biology, 6*(5), 430-440.

Zhong, M., Yue, L., Liu, W., Qin, H., Lei, B., Huang, R., . . . Kang, Y. (2023). Genome-wide identification and characterization of the polyamine uptake transporter (Put) gene family in tomatoes and the role of Put2 in response to salt stress. *Antioxidants, 12*(2), 228.

Zhong, M., Yue, L., Qin, H., Wang, G., Xiao, L., Cheng, Q., . . . Kang, Y. (2023). TGase-induced Cd tolerance by boosting polyamine, nitric oxide, cell wall composition and phytochelatin synthesis in tomato. *Ecotoxicology and Environmental Safety, 259*, 115023.

Zhang, Y., Wu, R., Qin, G., Chen, Z., Gu, H., & Qu, L. J. (2011). Over‐expression of WOX1 Leads to Defects in Meristem Development and Polyamine Homeostasis in Arabidopsis F. *Journal of integrative plant biology*, 53(6), 493-506.

Zhu, J.-K. (2002). Salt and drought stress signal transduction in plants. *Annual review of plant biology, 53*(1), 247-273.

Zhu, M., Shabala, L., Cuin, T. A., Huang, X., Zhou, M., Munns, R., & Shabala, S. (2016). Nax loci affect SOS1-like Na^+^/H^+^ exchanger expression and activity in wheat. *Journal of Experimental Botany, 67*(3), 835-844.

**Supplementary figures**


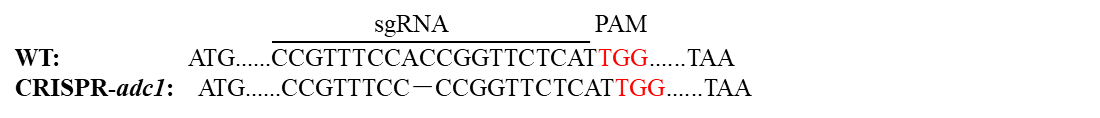


**Figure S1**. Generation of *adc1* mutant by CRISPR-Cas9 system. Black horizontal line indicates sgRNA. Red font indicates protospacer-adjacent motif (PAM) sequences, and black dash indicates deletion. The *adc1* line has a 1 bp deletion, leading to a premature termination of the ADC protein.


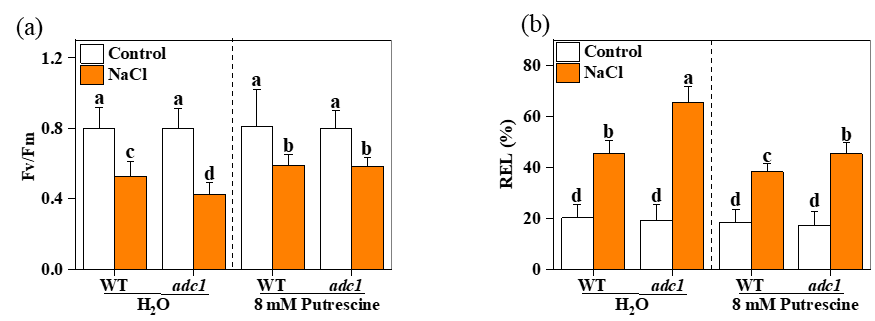


**Figure S2**. The Fv/Fm (a) and REL (b) in WT and *adc1* mutant after exogenous putrescine treatment under salt stress. Data are presented as mean values±SD; n=3. Different letters indicate signiﬁcant differences between treatments (*P*<0.05, Duncan’s multiple range test). At least three independent experiments were performed.


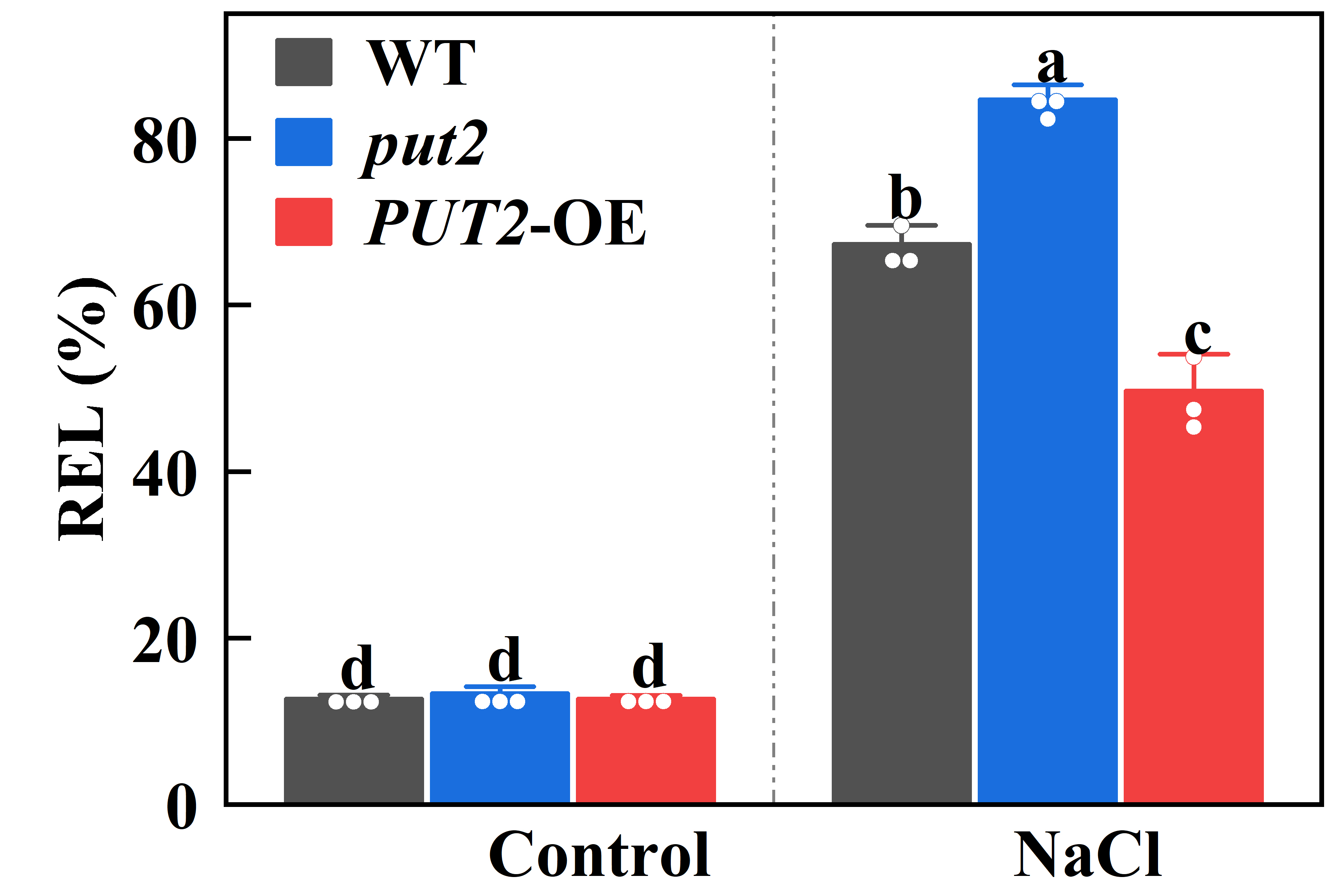


**Figure S3**. The REL in WT, *put2* mutant and *PUT2*-OE plants exposed salt treatment for 7 days. Data are presented as mean values±SD; n=3. Different letters indicate signiﬁcant differences between treatments (*P*<0.05, Duncan’s multiple range test). At least three independent experiments were performed.


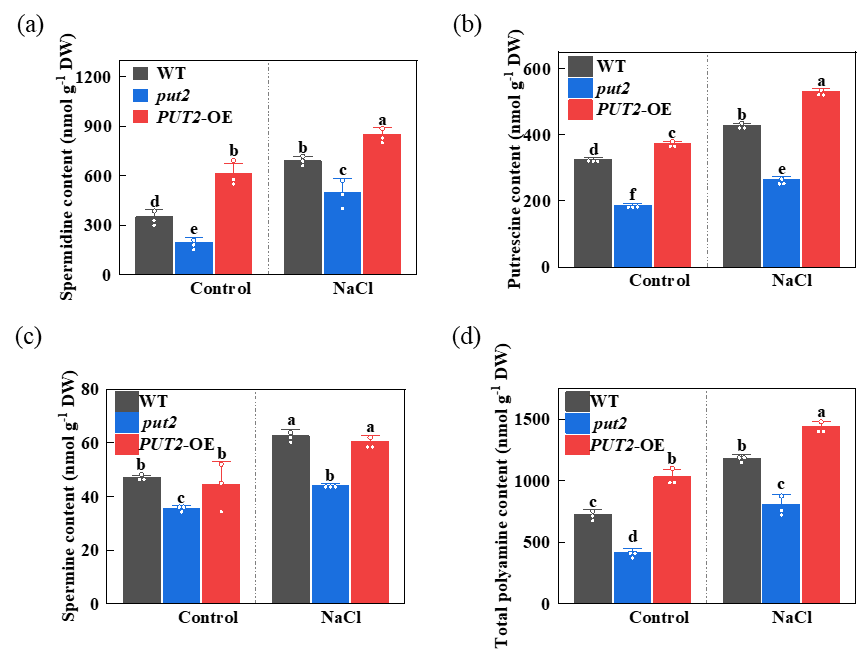


**Figure S4**. The spermidine (a), putrescine (b), spermine (c), and total polyamines content in WT, *put2* mutant and *PUT2*-OE plants exposed salt treatment for 7 days. Data are presented as mean values±SD; n=3. Different letters indicate signiﬁcant differences between treatments (*P*<0.05, Duncan’s multiple range test). At least three independent experiments were performed.


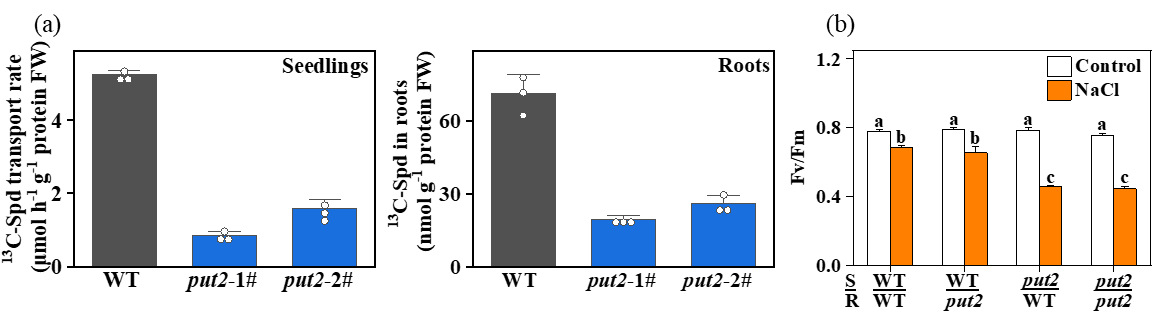


**Figure S5**. Spermidine uptake assays in the *put2* mutant and WT plants (a). The Fv/Fm in grafted plants with *put2* as rootstock or scion exposed salt treatment for 7 days (b). Data are presented as mean values±SD; n=3. Different letters indicate signiﬁcant differences between treatments (*P*<0.05, Duncan’s multiple range test). At least three independent experiments were performed.


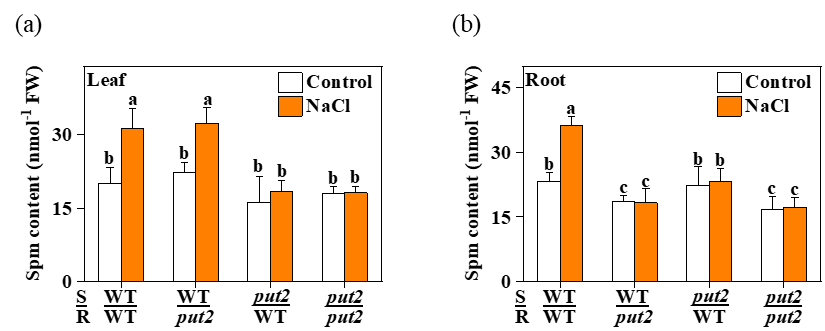


**Figure S6**. The spermine (Spm) accumulation in leaf (a) and root (b) in grafted plants with *put2* as rootstock or scion. Data are presented as mean values±SD; n=3. Different letters indicate signiﬁcant differences between treatments (*P*<0.05, Duncan’s multiple range test). At least three independent experiments were performed.


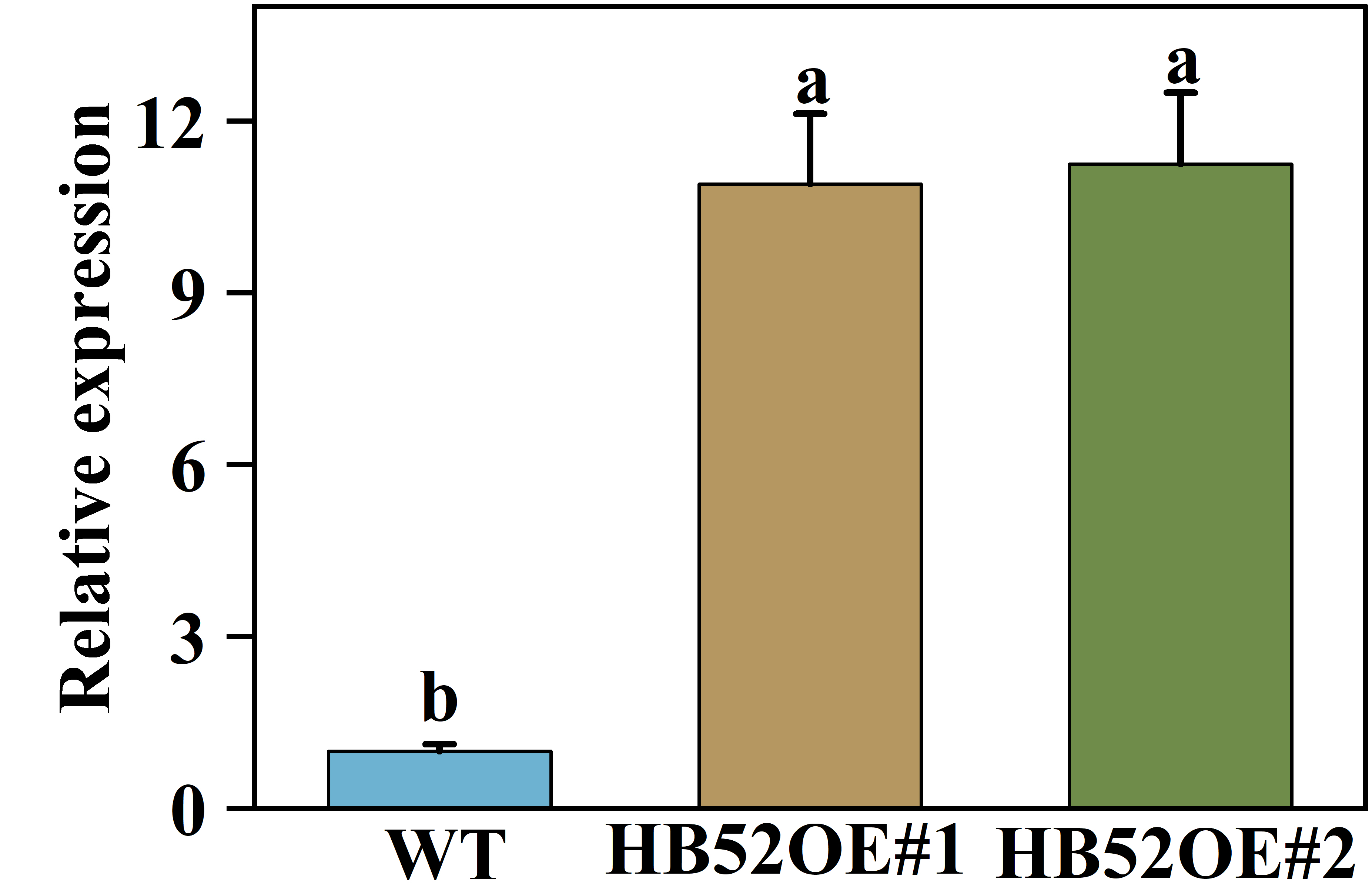


**Figure S7**. Identification of *HB52* overexpression lines by qRT-PCR. Data are presented as mean values±SD; n=3. Different letters indicate signiﬁcant differences between treatments (*P*<0.05, Duncan’s multiple range test). At least three independent experiments were performed.


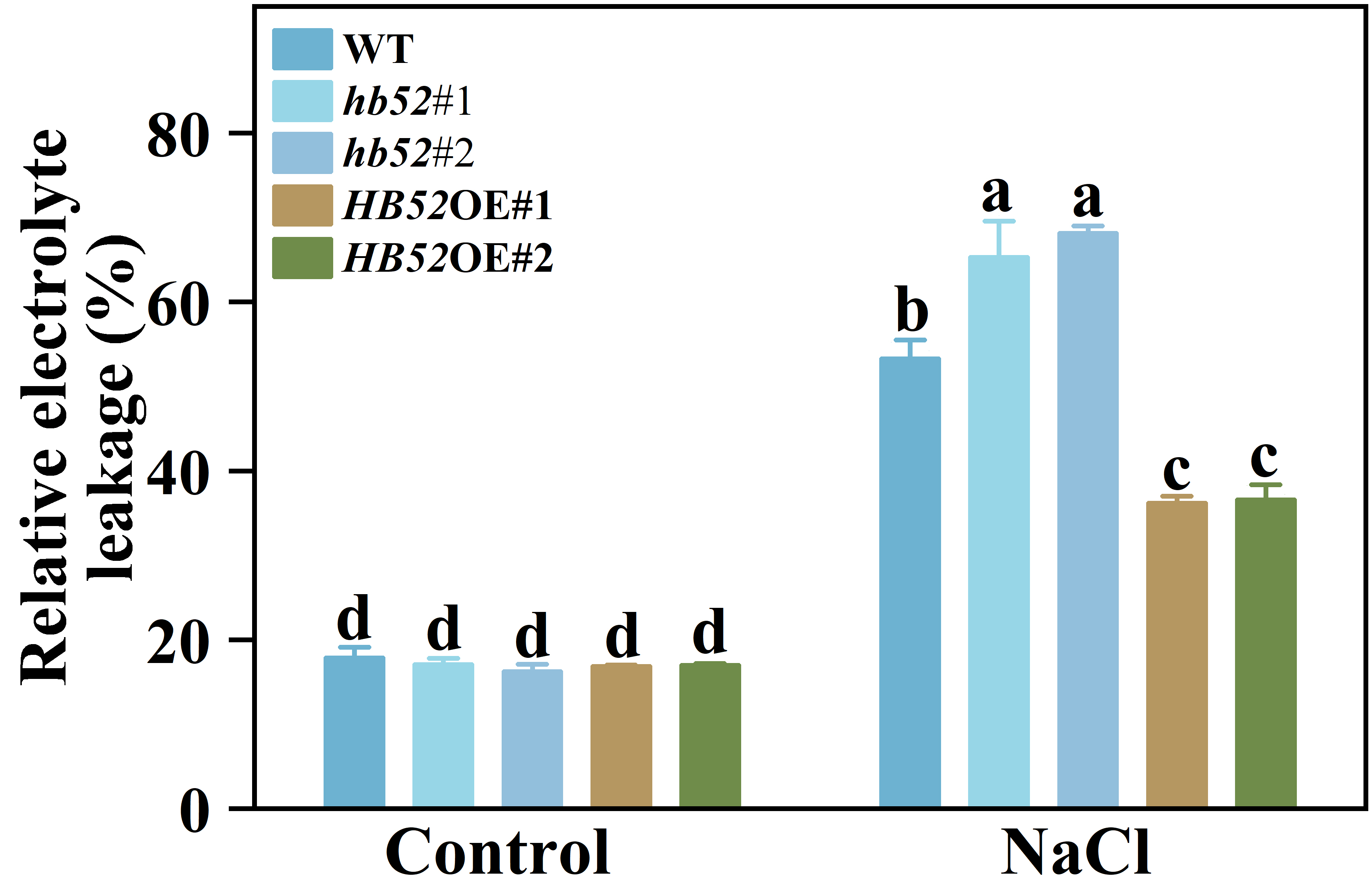


**Figure S8**. The REL in WT, *hb52* mutant and *HB52*-OE plants exposed salt treatment for 7 days.

Data are presented as mean values±SD; n=3. Different letters indicate signiﬁcant differences between treatments (*P*<0.05, Duncan’s multiple range test). At least three independent experiments were performed.


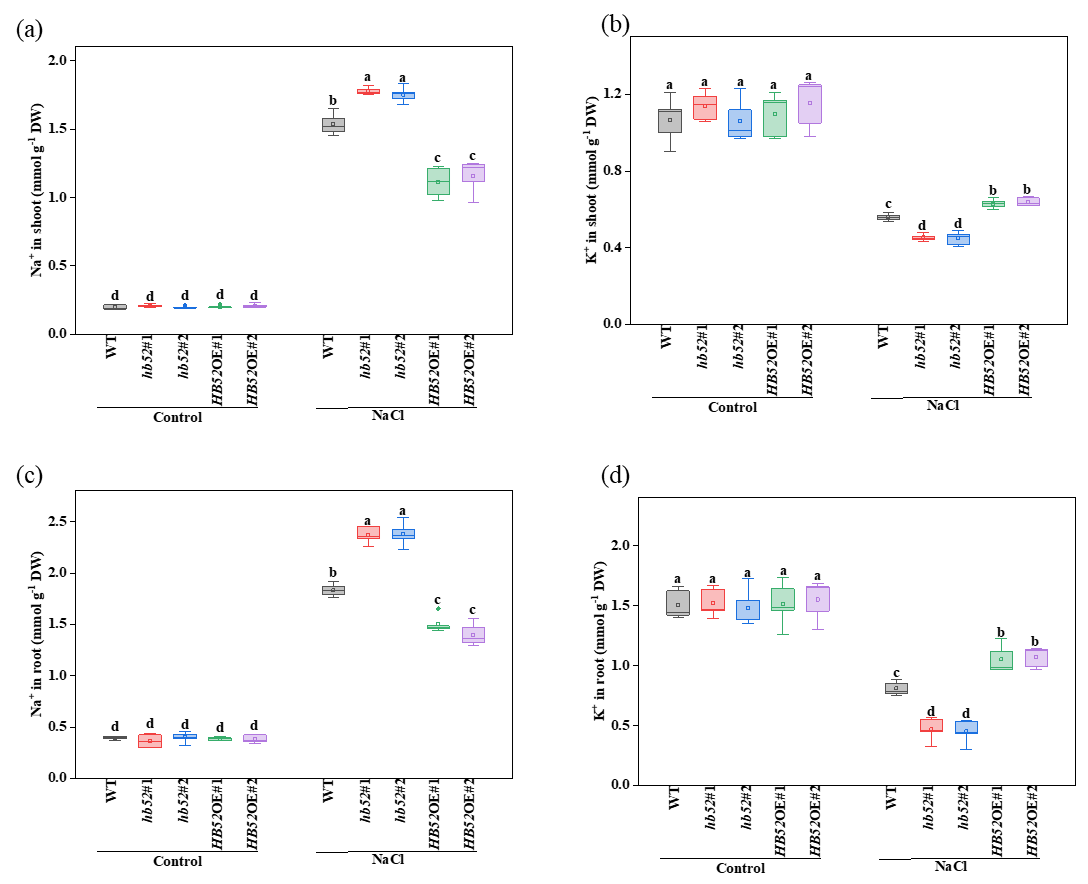


**Figure S9**. **Ion content in the shoots and roots of different genotypes.** Plots showing Na^+^ (a, c) and K^+^ (b, d) contents of shoots or roots of 4-week-old seedlings of the indicated genotypes treated with or without 125 mM NaCl for 5 d (n=5 plants per genotype), respectively. Significant differences between WT and other genotypes were evaluated by two-way ANOVA, followed by Duncan’s multiple range test. DW, dry weight.


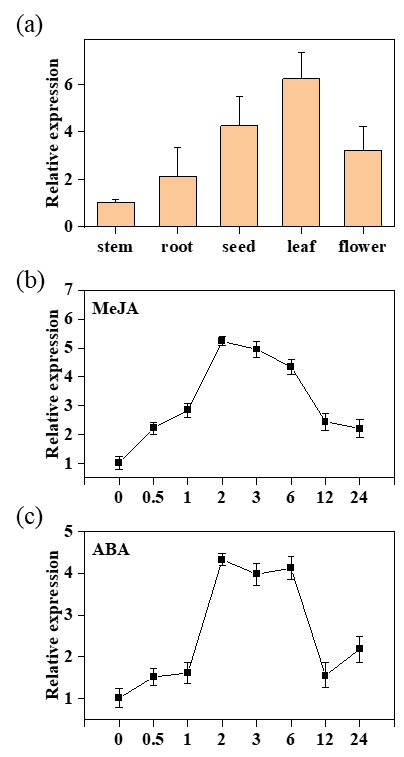


**Figure S10**. **Expression pattern of *HB52* and response to abiotic stresses.** (a) qRT-PCR results showing the expression of *HB52* in different plant tissues, including stem, root, seed, leaf, flower. (b and c) Transcript level of *HB52* in response different phytohormones. 3-week-old wild type seedlings were separately with 10 MeJA or 10 μM ABA for 0, 0.5, 1, 2, 3, 6, 12, 24 h. The experiment was repeated 3 times. Data are means ± SD (n = 3). *Actin* was used as a reference gene. Different letters indicate signiﬁcant differences between treatments (*P*<0.05, Duncan’s multiple range test). At least three independent experiments were performed.


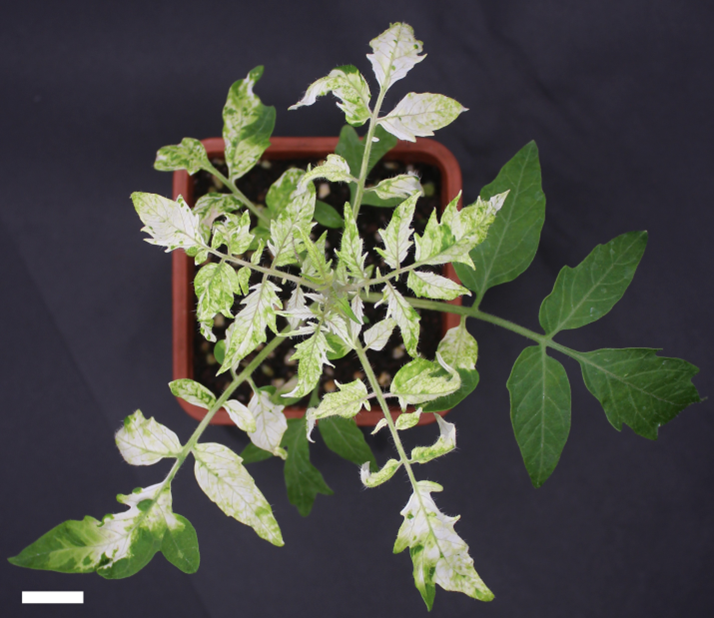


**Figure S11**. Phenotypes for the silencing of *PDS* in WT background, Bar=5 cm.
